# Implanted microelectrode arrays in reinnervated muscles allow separation of neural drives from transferred polyfunctional nerves

**DOI:** 10.1038/s41551-025-01537-y

**Published:** 2025-10-24

**Authors:** Laura Ferrante, Anna Boesendorfer, Deren Y. Barsakcioglu, Benedikt Baumgartner, Yazan Al-Ajam, Alexander Woollard, Norbert V. Kang, Oskar C. Aszmann, Dario Farina

**Affiliations:** 1 Department of Bioengineering, Imperial College London, London, UK; 2 Clinical Laboratory for Bionic Extremity Reconstruction, Department of Plastic, Reconstructive and Aesthetic Surgery, Medical University of Vienna, Vienna, Austria; 3 Plastic Surgery Department, Royal Free Hospital NHS Trust, London, UK; 4 Department of Plastic, Reconstructive and Aesthetic Surgery, Medical University of Vienna, Vienna, Austria

**Keywords:** Computational neuroscience, Motor neuron, Machine learning, Biomedical engineering, Microarrays

## Abstract

Targeted muscle reinnervation surgery reroutes residual nerve signals into spare muscles, enabling the recovery of neural information through electromyography (EMG). However, EMG signals are often overlapping, making the interpretation of limb functions complicated. Regenerative peripheral nerve interfaces surgically partition the nerve into individual fascicles that reinnervate specific muscle grafts, isolating distinct neural sources for precise control and interpretation of EMG signals. Here we combine targeted muscle reinnervation surgery of polyvalent nerves with a high-density microelectrode array implanted at a single site within a reinnervated muscle, and via mathematical source separation methods, we separate all neural signals that are redirected into a single muscle. In participants with upper-limb amputation, the deconvolution of EMG signals from four reinnervated muscles into motor unit spike trains revealed distinct clusters of motor neurons associated with diverse functional tasks. Our method enabled the extraction of multiple neural commands within a single reinnervated muscle, eliminating the need for surgical nerve division. This approach holds promises for enhancing control over prosthetic limbs and for understanding how the central nervous system encodes movement after reinnervation.

## Abstract (web summary)

Following amputation, the neural signals embedded in electromyography recordings from a high-density microelectrode array inserted in a reinnervated muscle can be functionally separated via a mathematical approach without surgically partitioning the distinct neural sources.

## Main

The central nervous system controls movements of the upper limb through the coordinated activity of hundreds of thousands of motor and sensory nerve fibres. Amongst these, thousands of α-motor neurons carry the neural signals (motor commands) from the spinal cord to the skeletal muscles^1^. In peripheral nerves, the axons of motor neurons are organized into fascicles. Nerves can be classified on the basis of the number of fascicles they contain^2^. Monofascicular nerves have a single fascicle, oligofascicular nerves contain a few fascicles and polyfascicular nerves have many fascicles. This classification reflects the internal organization and complexity of the nerve structure as well as the number of functions that the nerve can encode (that is, polyvalent nerves). Amputation of the upper extremities disrupts the upper-limb motor units and thus the communication between the central nervous system and the periphery. The motor neurons previously innervating the missing limb are left without target muscles to innervate. However, neural signals for muscle control continue to travel through the nerves even after amputation. Targeted muscle reinnervation (TMR) involves the transfer of polyfascicular nerves, which previously innervated muscles of the missing limb, to surgically denervated target muscles^3^. The target muscle acts as a biological amplifier for the neural signals transmitted by the rerouted nerve and allows these signals to be captured through standard human–machine interfaces using electromyography (EMG). A pioneering study^4^ reported that the difference in innervation ratios of the original and targeted muscles causes the motor neurons to compete to reinnervate the available muscle fibres when the donor nerve has an axonal surplus. Although a relatively small percentage of transferred motor neurons may survive post-TMR, Bergmeister et al.^5^ found no loss of muscle force generation in animal models of TMR and a 1.7-fold increase in the innervation ratio (that is, hyper-reinnervation) of the targeted muscle. Subsequent histological studies demonstrated that hyper-reinnervation led to a greater number of small motor units and to a shift in the composition of the host muscle to accommodate the rerouted nerve^6^. The same authors^7^ hypothesized that such hyper-reinnervation determines a heterogeneous distribution of groups of motor units that may be independently controlled, thereby potentially creating a multidegree of freedom neural biointerface for prosthesis control. Although a polyfascicular nerve supports extensive cognitive control and numerous functions that (in principle) can be decoded from the resulting EMG signals, decoding the neural signals associated with these multiple functions has proven to be a substancial challenge. Classic TMR surgery transfers multiple polyfascicular nerves into distant portions of the same muscle or of different muscles to avoid interference between the recorded signals^3,8–10^. Surface EMG sensors are typically positioned over multiple, spatially distinct reinnervation sites, where global EMG features are analysed to decode motor commands, generating a single control signal for each targeted reinnervation area. This contrasts with the multiple neural signals reaching the reinnervated muscle through a single polyfascicular nerve.

Regenerative peripheral nerve interfaces (RPNIs) have been proposed for enhancing signal specificity and extracting functionally separate neural signals transmitted through polyfascicular nerves. This is achieved by surgically separating the fascicles of a nerve and rerouting each one into a separate muscle graft, forming an RPNI unit^11^. Each RPNI unit is monitored via a bipolar intramuscular EMG. Although the donor nerve fascicles are separated without prior knowledge of their specific functional roles, a postsurgical assessment determines whether each RPNI can generate an independent EMG signal. Consequently, RPNIs offer the potential to provide multiple independent signals for controlling several degrees of freedom or tasks in a prosthetic device from a single polyfascicular nerve^11–14^.

RPNIs with chronic implants have demonstrated signal stability for over a year^11,15^. However, this approach has limitations that may affect the efficacy of the nerve transfer procedure^16,17^. First, the nerve is divided into fascicles without prior knowledge of the functional role of each. This may result in fascicles and, therefore, RPNI units not providing a usable control signal. Second, detecting EMG signals from each RPNI unit remains challenging outside a laboratory setting. Third, the number of fascicles, and thus the potential number of independent signals, is limited by the need to attach each fascicle to a muscle graft. Finally, these fascicles are sutured to a small segment of muscle not respecting the neuromuscular entry zone and thus run the risk of not making any functional connections. In such cases, RPNI units are lost while the surgery results in permanent damage of the nerve.

**Fig. 1:**
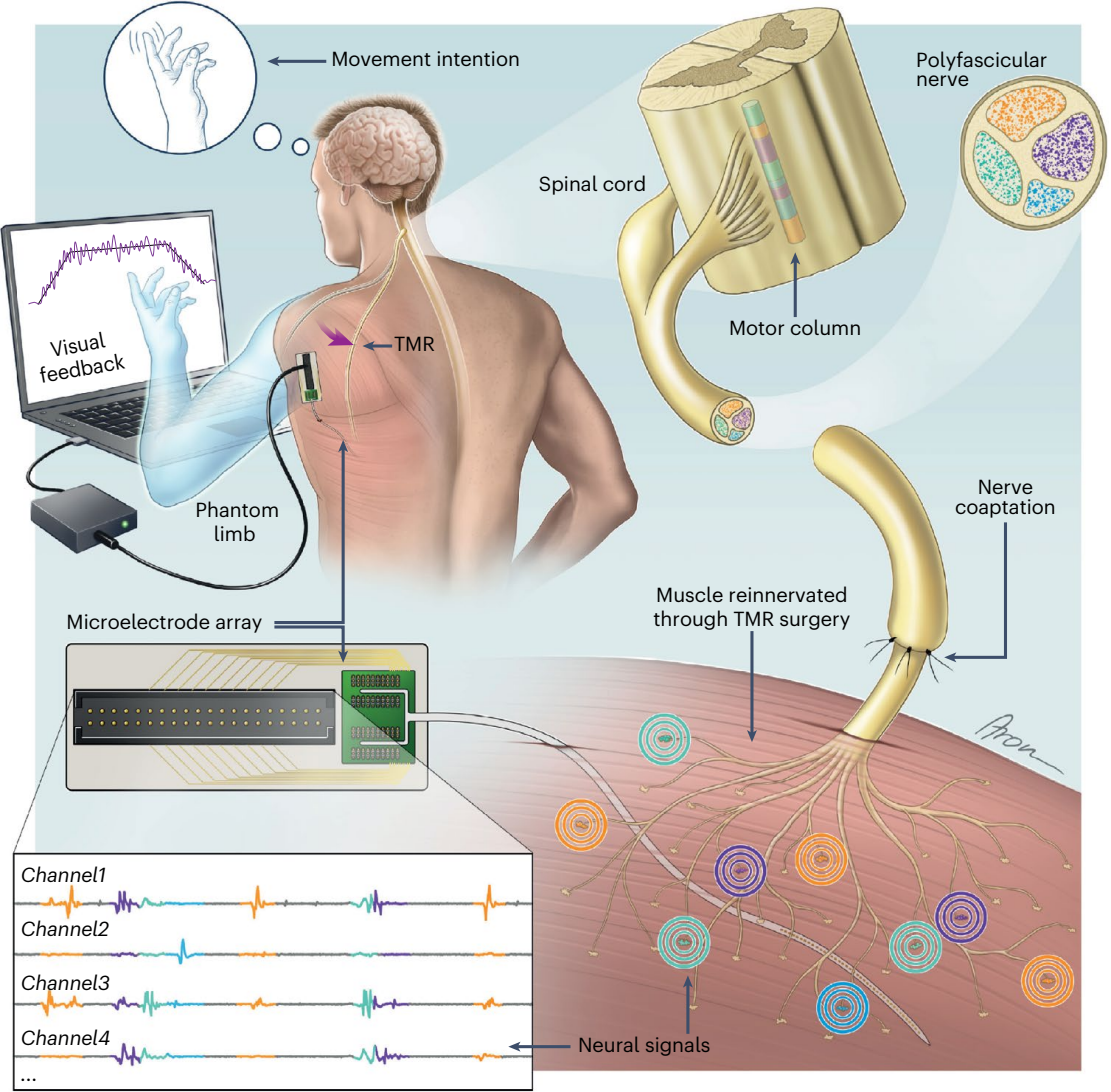
Biointerface based on TMR of a polyfascicular nerve and the use of a microelectrode array for recording and decoding. Left: a glenohumeral patient has undergone TMR nerve transfer surgery: a polyfascicular nerve that previously innervated multiple upper-limb muscles (shaded blue) was transferred (yellow nerve indicated by purple arrow) into a spare targeted muscle. The participant was asked to perform different tasks with his missing limb (for example, index finger extension) while the intramuscular activity of the reinnervated muscle was recorded using a 40-channel microelectrode array^19^. The 40 EMG channels are distributed over 2 cm, and each electrode has a diameter of 140 μm (for comparison, muscle fibres have diameters in the range 10–100 μm). The participant had real-time visual feedback of the median EMG activity (purple signal) that had to be modulated to match a target trapezoidal profile displayed on the screen. This resulted in a contraction of the reinnervated muscle. Right: the schematic provides insight into the reinnervation following TMR surgery. The axons of the rerouted neuron innervate fibres of the targeted muscles, creating a heterogeneous distribution of motor units (MUs). The activity of MUs is recorded in vivo by the intramuscular array. From the intramuscular recordings, the individual activity of the MUs can be extracted by blind-source separation methods. Here we hypothesize that the heterogeneous distribution of MUs corresponds to a functional organization of MUs. Thus, clusters of MUs associated with different tasks of the missing limb can be identified. Note that the neural structures, muscle fibres and microelectrode array are not represented with an accurate scaling to improve clarity. Credit: illustration provided by Aron Cserveny.

Here, we hypothesize that neural signals carried by a polyfascicular nerve to a reinnervated muscle can be functionally separated using high-density microelectrode arrays and a mathematical approach, avoiding the need to surgically separate nerve fascicles to create anatomically separated sites of EMG activity. With this paradigm, the vast array of functional properties of any polyvalent nerve will be displayed polytopically in a large well vascularized muscle. This would combine the benefits of both the quantity of signals with RPNIs and the robust signal production with the vascularized muscle reinnervation of TMR. To evaluate this hypothesis, we recorded high-density intramuscular EMG activity of four reinnervated muscles from three volunteers while they performed several tasks with their missing limb and decomposed the EMG signals into their constituent motor unit activities. This concept is illustrated in Fig. 1 for an exemplar task of index finger extension. We investigated whether the mixture of neural signals carried by the multiple fascicles of the nerve could be mathematically disentangled into a higher-dimensional neural space. Furthermore, since it is well known that, during motor task execution, motor neurons are clustered into functional groups by the projection of common synaptic input^18^, we investigated the degree of correlation in motor unit activities and employed a dimensionality-reduction method to identify the effective dimensionality of the neural manifold underlying the activity of the reformed motor units.

## Results

### Motor unit recordings in reinnervated muscles

Three men (aged 62, 34 and 52 years) who suffered from an amputation of their limb at the transhumeral (patient (P)1 and P2) and glenohumeral level (P3) were included in this study. They underwent their TMR surgery 7–12 years before the experiments, to treat neuroma and phantom limb pain (P1) and to improve prosthesis control (P2 and P3). They all used a myoelectric prosthesis regularly and had good phantom sensation in their absent limbs. Detailed patient characteristics are reported in Table 1. A total of four reinnervated muscles were examined: three (TMR1, TMR2 and TMR3) were reinnervated by the ulnar nerve and one (TMR4) by the radial nerve.

**Table 1:** Patient characteristics, surgery details, prosthetic information and experimental protocol

TMR volunteers	P1	P2	P3
Patient characteristics			
Age	62 years	34 years	52 years
Time since amputation	44 years	9 years	19 years
Level of amputation	Transhumeral	Transhumeral	Glenohumeral
Surgery details			
Time since TMR surgery	7 years	8 years	12 years
TMR site—innervating nerve	Pectoralis minor—ulnar Pectoralis major abdominal pars—median	Biceps short head—ulnar Brachialis—median Triceps lateral head—split deep radial branch Brachioradialis—split deep radial branch	Pectoralis major clavicular pars—musculocutaneus Pectoralis minor—ulnar Pectoralis major sternocostal pars—median pars I (lateral) Pectoralis major abdomial pars—median pars II (medial) Latissimus dorsi/teres major—radial
Prosthetic information			
Prosthetic type	Myoelectric	Myoelectric	Myoelectric
Prosthetic use	15 h per day	2–12 h per day for 2 days per week	0–12 h per day for 1–2 days per week
Number of used EMG sensors	2	4	6
Movements for prosthetic control	Elbow flexion and elbow extension	Tripod, fingers extension, elbow flexion and elbow extension	Tripod, fingers extension, elbow extension, elbow flexion and pronation and supination
Experimental protocol			
Insertion location—microelectrode 1	Pectoralis minor—ulnar (TMR1)	Biceps short head—ulnar (TMR2)	Pectoralis minor—ulnar (TMR3)
Movements associated with microelectrode 1	Ulnar deviation Pinky flexion Pinky abduction Intrinsic position (MCP flexion) Fist/flexion of fingers Wrist flexion	Ulnar deviation^−^ Pinky flexion Intrinsic and thumb adduction Fist/flexion of fingers	Ulnar deviation* Pinky flexion Pinky abduction* Intrinsic position (MCP flexion) Thumb adduction
Insertion location—microelectrode 2			Latissimus dorsi—radial (TMR4)
Movements associated with microelectrode 2			Supination Wrist extension^−^ Finger extension Thumb extension Index finger extension^−^ Pinky extension
Protocol	Four reps per movement Ramps: 4 s Isometric: 10 s at 10% MVC	Six reps per movement Ramps: 2 s Isometric: 5 s at 20% MVC	Six reps per movement Ramps: 2 s Isometric: 5 s at 10% MVC *Ramp: 4 s Isometric: 5 s at 20% MVC

MCP, metacarpophalangeal joint. The superscript minus sign denotes a comment of patients that the movements were difficult to imagine for the patient. The asterisk denotes that for these movements, a different protocol (see *) was applied.

We used a single implant for each examined reinnervated muscle to obtain a highly dense sample of intramuscular EMG activity. Each implanted microelectrode array consisted of 40 recording sites, linearly distributed with an interelectrode distance of 500 μm over 2 cm) of overall length^19^. Blind-source separation was used to decode the multichannel array recordings into the activities of individual motor units. This invasive biointerface allowed us to overcome the limitations of non-invasive sensing and to obtain a high yield of decoded motor units^6^. The current study represents the first exploration of such implanted technology for a human–machine interface paradigm in patients with TMR. Prior work has evaluated the feasibility of this approach in animal models of TMR^20^.

An example of experimental setup and protocol is shown in Fig. 1 for index finger extension performed by P3. Participants performed different tasks of their missing limb, which resulted in contractions of their reinnervated muscle. The task-specific EMG amplitude at the maximum voluntary contraction (MVC) was recorded and used to normalize subsequent EMG signals for the same task. Participants received auditory guidance from the experimenters when performing their tasks, as well as real-time visual feedback of the reinnervated muscle EMG activity (purple signal) and the targeted contraction profile they had to match (black line). The visual feedback was the bipolar signal obtained from selecting two channels of the microelectrode array that resulted in the maximum signal amplitude. Depending on the muscle and reinnervation achieved by the TMR surgery, different missing limb movements were included in the protocol for each participant, ranging from single (for example, index finger extension) to a combination of degrees of freedom tasks (for example, tripod grasp); each task was repeated four times by P1 and six times by P2 and P3. The protocol for each participant is detailed in Table 1 and discussed in the ‘Experimental protocol’ section in the Methods. It is important to note that the EMG data for each task were normalized according to the task-specific MVC, meaning that the absolute amplitude of EMG signals at, for example, 20% of MVC for a specific task might differ from the 20% MVC of another task. As a result, the recruitment and discharge properties of motor units varied considerably across tasks.

**Fig. 2:**
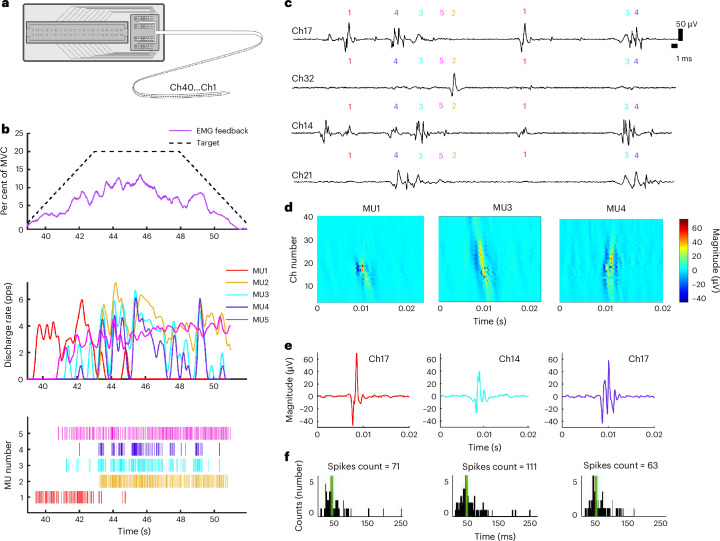
Characteristics of MUs in targeted muscles reinnervtaed by polyfascicular nerves. **a**, A microelectrode array containing 40 EMG channels (Ch) is implanted into each examined reinnervated muscle to record high-density intramuscular EMG activity. **b**, In an exemplar task repetition, participant P3 contracts the reinnervated muscle TMR4 (Table 1) while performing index finger extension with his missing limb. Top: a bipolar signal was derived from channels of the microelectrode array that resulted in the maximum amplitude and was used as visual feedback of reinnervated muscle activity (purple signal). The participant modulated the muscle contraction to match the target EMG activity (black dotted line; trapezoid contraction up to a percentage of task-specific MVC). Middle: five motor units (MUs) were reliably decomposed from the intramuscular recordings in this example. The smoothed discharges obtained by low-pass filtering the instantaneous firing rate of each MU with a Hanning window of 400 ms are shown. Bottom: instantaneous discharges of active MUs are depicted with vertical lines, each indicating a MU discharge at a given time instant. The spike trains and smoothed discharges of MUs have the same colour in the two plots. **c**, EMG voltages for active MUs on different channels of the microelectrode array. The discharge time of MUs is indicated with a colour-coded number on top of each MUAP in the signal. A channel records a MUAP with an amplitude that depends on the position of the detection site with respect to the fibres innervated by the MU. For this reason, the MUAP waveform differs across channels. **d**, A two-dimensional image of the average (across all firing instances) MUAP distribution along the 40 channels is shown for some exemplar MUs. MUs had different morphologies, as indicated by the potentials spanning a few or almost all channels of the microelectrode array. **e**, Average MUAPs of the MUs on the channel where each MUAP had maximum peak-to-peak amplitude. **f**, The distribution of intervals between MU discharges for the same MUs depicted in **e**, using a 1-ms bin size. Credit: illustration in panel **a** provided by Aron Cserveny.

The microelectrode array recorded high-quality signals in all cases (Fig. 2a,c), with average root mean square of the baseline noise across channels below 8 μV (5.3 ± 0.4 μV TMR1, 7.8 ± 0.4 μV TMR2, 5.7 ± 0.8 μV TMR3, 7.8 ± 0.2 μV TMR4). Figure 2c shows a sample of intramuscular signals from exemplar channels of the microelectrode array. The motor unit activities can be observed from these multiunit recordings. The EMG signals were decomposed into spike trains (Fig. 2b) of active motor units using the blind-source-separation method presented in Muceli et al.^19^ and subsequently inspected with the spike-sorting interactive software EMGLAB^21^ (see ‘Motor unit decomposition’ in the Methods for details). Figure 2d shows examples of the distribution of the average electrical potential of the motor units across the 40 channels of the microelectrode array, revealing motor units with different morphology and territory. In this example, MU1 spans about ten channels, while the action potential of MU2 is distributed across tens of channels, revealing a larger territory. The average values of the morphological and discharge properties of the identified motor units are reported in Supplementary Table 1 for all the tasks performed by the reinnervated muscles.

### Motor unit activity

Two fundamental mechanisms underlie the coordinated control of motor units: motor units within a motor unit pool are recruited in an orderly manner^22^ according to the motor neuron size (that is, the surface area of the soma and dendrites), from smaller to larger motor units^23^; a gradual increase in force is achieved by recruiting new motor units and concurrently by modulation of the firing rate of the active motor units (rate coding)^24,25^.

#### Motor unit count and satellite potentials

A total of 111 motor units were identified from EMG signals recorded from the four examined reinnervated muscles when considering all the tasks, with a pulse-to-noise ratio >30 dB (ref. ^26^). Motor units observed in <70% of the repetitions were not included in the motor unit count and were not used in subsequent analyses since their detection was not considered sufficiently consistent. The number of removed motor units was 9, 12, none and one for each reinnervated muscle, considering all the recorded tasks. The average number of identified motor units across tasks was 7.6 ± 0.8, 4.6 ± 1.1, 3.1 ± 1.4 and 3.8 ± 1.5 for the four reinnervated muscles. About a third of the motor units showed action potentials with a satellite potential, distinct from the main potential but time-locked to it^27^. These potentials occurred from 8.8 to 32 ms from the main potential peak. A waveform was considered a satellite of an earlier occurring potential waveform if it appeared >80% of the time concurrently with the main potential, with a constant delay. An example of satellite potential is shown in Supplementary Fig. 2.

#### Discharge characteristics

The distribution of the interspike intervals (ISI) of the motor unit discharges, during the plateau part of the contractions, was tested for normality. A long ISI (>250 ms) were removed, as these were probably due to pauses in the tonic activity of motor unit firing. As shown in Fig. 2f, the majority of the ISI of the analysed motor units had skewness and kurtosis that deviated significantly from a normal distribution according to the D’Agostino–Pearson test^28^. The average percentages of normally distributed ISI considering all the units per volunteer were 27.8% ± 14.7%, 38.7% ± 18.9% and 22.6% ± 13.5% for P1, P2 and P3, respectively.

The median firing rate (MFR) of the motor units was computed as the median of the inverse of the ISI of discharges during the plateau phase of the contraction. The average MFR across tasks was 12.4 ± 3.3, 13.4 ± 3.9, 16.1 ± 4.2 and 18.4 ± 4.1 for TMR1, TMR2, TMR3 and TMR 4, respectively. TMR1 and TMR2 had a significantly different distribution of MFR than TMR3 and TMR4 (*P* < 0.05 in all cases); the test for TMR1 and TMR2 did not reject the null hypothesis (*P* = 0.3), while TMR3 and TMR4 showed a significantly different distribution of MFR (*P* = 0.006).

The coefficient of variation (CoV) of the ISI, calculated as the standard deviation of the ISI divided by the median ISI, was used to measure the variability in the motor unit discharge. The average (across tasks) CoV of motor unit spike trains varied considerably across reinnervated muscles, with values ranging from 0.2% ± 0.1% (intrinsic hand task for TMR1) to 0.1% ± 0.1% (wrist extension for TMR4). The average CoV across tasks was 21.7% ± 11.3%, 28.5% ± 10.7%, 37.4% ± 12.9% and 42.8% ± 17.6% for the four reinnervated muscles, respectively. TMR1 and TMR2 distribution of CoV significantly differed from that of TMR3 and TMR4; TMR3 and TMR4 did not differ from each other (*P* = 0.58). The CoV values for TMR3 and TMR4 were higher than those observed in physiologically innervated muscles during muscle contractions, probably due to the quality of the visual feedback (EMG instead of force) and the difficulty in generating motor commands. Especially for TMR3 and TMR4, there were cases where the firing pattern of motor units exhibited irregularities (for example, in Fig. 2b, bottom), such as intermittent firing throughout the contraction. This might have caused an underestimation of the MFR and larger values of CoV. Despite large discharge variability indicated by the CoV, the MFR were within the physiological range reported for voluntary contractions of physiologically innervated muscles (minimum 5–7 pulses per second, maximum 40 pulses per second for moderate isometric contractions (>30% of MVC)^29^. The average task-specific values and the average values of MFR and CoV per muscle (across the different tasks) are reported in Supplementary Table 1.

We observed cases where later recruited motor units were the last to be derecruited, similar to unusual motor unit behaviour previously reported in elderly individuals^30^. An example is the index extension task for TMR4, shown in Fig. 2b; MU1 is the first to be recruited, but it stops firing during the plateau phase at a higher level of MVC. MU2 is recruited during the constant phase but is derecruited last, at a lower MVC value. These behaviours were observed consistently across repetitions of tasks. Finally, there were cases where motor units were consistently recruited only at the beginning of the contraction (ramp-up phase) (TMR4, extension of fingers; TMR1, intrinsic) but were not active in the other phases of the contractions.

### Motor unit tracking

#### Shared and task-specific motor units

**Fig. 3:**
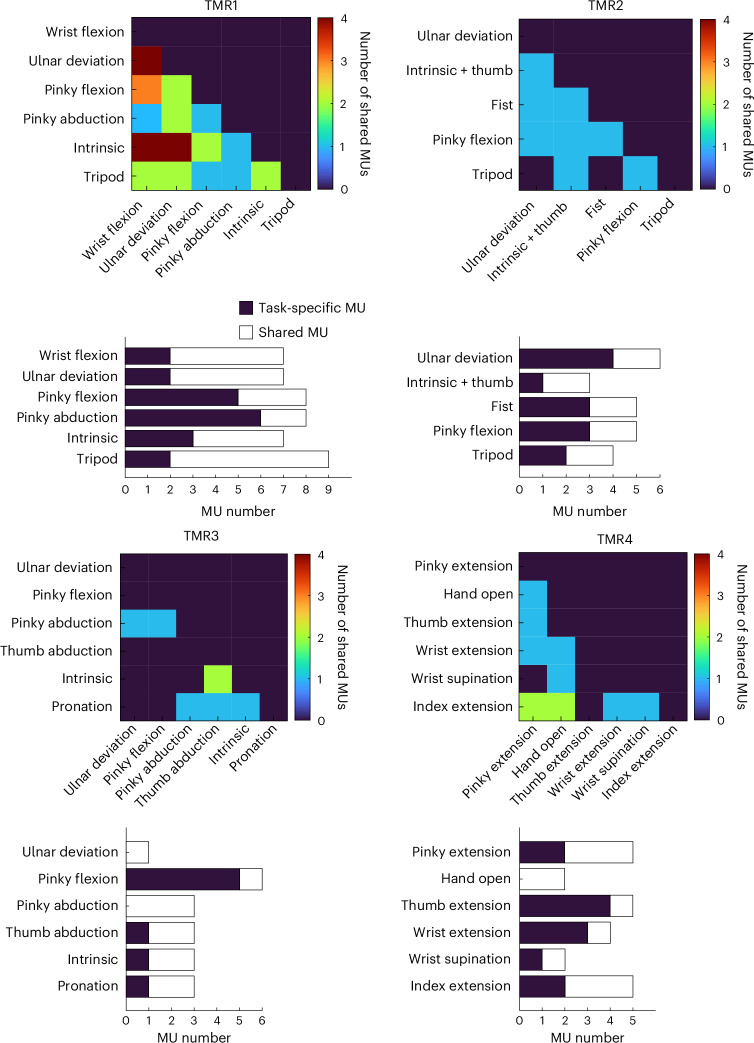
Tracking of MUs across EMG signals recorded for different tasks of the missing limb revealed task-specific and shared MUs. For each reinnervated muscle, the diagrams at the top detail the relation between tasks of the missing limb in terms of the number of shared motor units (MUs). The bar plots report the total number of MUs identified per task, with the proportion of task-specific and shared MUs highlighted in white and blue, respectively. Only MUs that could be accurately decomposed and were active in ≥70% of task repetitions were considered.

For each reinnervated muscle, motor units identified during a task of the missing limb were tracked across other tasks to determine whether they were independent (that is, task-specific motor units) or contributed to multiple tasks (that is, shared motor units). The average motor unit action potential (MUAP) waveform across channels obtained by spike-triggered averaging in a 20-ms window was used to track the motor units (see ‘Motor unit tracking across tasks’in the Methods for details). The count of task-specific and shared motor units is reported in Fig. 3; diagrams in the same figure detail which tasks were performed with shared motor units. A total of 23 tasks were recorded considering all reinnervated muscles, 6 tasks for each of three reinnervated muscles (TMR1, TMR3 and TMR4) and 5 tasks for TMR2. Only 3 out of the 23 tasks lacked task-specific motor units. These were all tasks of TMR3 and TMR4 performed by the same participant P3 (ulnar deviation, pinky abduction and hand open); only a limited number of motor units could be decomposed for these tasks (1, 3 and 3 MUs, respectively). Furthermore, none of the tasks were performed using exclusively task-specific motor units. On average, the tasks of TMR1 to TMR4 had 43.3% ± 21.2%, 54.0% ± 13.0%, 30.6% ± 30.6% and 47.5% ± 28.9% task-specific units, respectively. This analysis included only motor units recruited in at least 70% of task repetitions.

#### Signal stability and motor unit specificity

We investigated the task specificity and stability of the signals throughout the experimental session by asking the participants to repeat some selected tasks (contractions at the same MVC) at the beginning (T1) and end (T2) of the experimental session, which lasted a few hours. We decomposed the EMG recording of T1 and T2 separately and then checked if the same motor units could be identified during both repetitions. For example, we asked P2 to repeat pinky flexion and tripod at the end of the experimental session, after repeated contractions of the other tasks. We hypothesized that the same motor units could be tracked during T1 and T2 if (1) the participants could formulate the motor command for the different tasks and execute them consistently and selectively and (2) no significant misplacement of the electrodes had occurred.

**Fig. 4:**
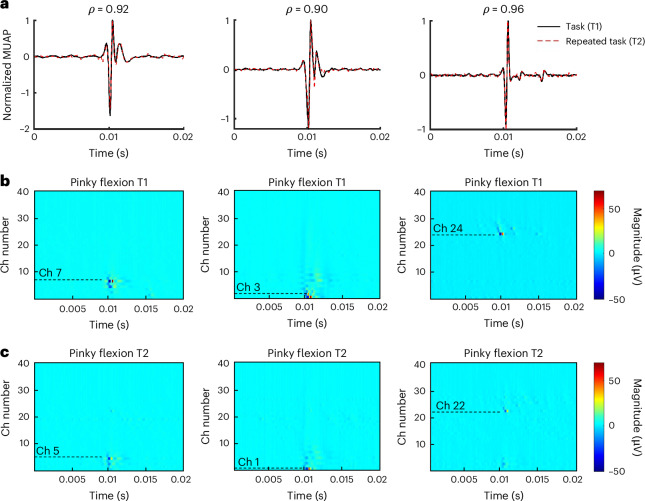
Tracking of MUs for tasks with significant time intervals between repetitions. **a**, An exemplar task, pinky flexion, performed by participant P2 at the beginning (T1) and the end (T2) of the experimental session. The EMG recordings at task repetitions T1 and T2 were decomposed separately to identify motor units (MUs). Among the five MUs recruited in T1, three could be tracked in T2. The average MUAP of the matched MUs is shown (black and red dotted line), and the goodness of the fit between the two is quantified by the coefficient of determination *ρ*. **b**, The average (across all firing instances per MU) distribution of three MUs action potential during T1 is shown. The dotted lines indicate the channel (Ch) at which the average MUAPs had maximum peak-to-peak amplitude. **c**, Corresponding average distribution of the MUs action potential is shown during T2. A consistent shift of two channels can be observed. In T2, the matched MUs are shifted towards channel 1, indicating a slight microarray displacement. The other two MUs identified during T1 could not be tracked in T2, possibly due to the electrode shift.

The microelectrode arrays inserted in TMR2 and TMR3 were inspected to confirm no obvious displacement of the electrodes had occurred due to cable pulling or unwanted motions. As detailed in the ‘Motor unit tracking across tasks’ in the Methods, the coefficient of determination between the MUAPs *ρ* ≥ 0.85 flagged a possible match between the two motor units. In addition, the motor unit territories were estimated from the distribution of the motor unit potential across channels and compared as an additional means of tracking motor units. All pairs of motor units flagged as a match by either approach were visually inspected by an expert examiner. These two approaches do not restrict the comparison with specific channels and can, therefore, track motor units even in the presence of electrode shift. Ambiguous cases, where the examined motor units had similar MUAP waveforms and were distributed in a single channel (for example, Fig. 4b) were resolved by assessing if the shift across channels was consistently detected in other tracked motor units. Three motor units could be tracked in T1 and T2 of pinky flexion. Matched motor units (in black and dashed red) are reported in Fig. 4a. In Fig. 4b,c, a consistent shift of two channels can be observed by examining the MUAP distribution of the three motor units. The motor unit detected in T1 had a maximum peak-to-peak amplitude at channel 7; however, this corresponded to a maximum peak-to-peak amplitude at channel 5 during T2, and the same shift was observed for the other two matched motor units. This indicated a slight displacement of the microelectrode array along the insertion direction.

All motor units recruited during T1 of tripod for P3, and flexion of fingers for P2 could be identified in T2. For pinky flexion performed by P3, four out of six motor units were detected in both repetitions of the tasks. The two unidentified motor units had MUAPs detected only in the first few channels. Overall, these results suggest that the participants executed the tasks consistently and selectively as several motor units were recruited during consecutive task repetitions and, notably, also during repetitions executed after significant time intervals.

### Estimation of neural drives from polyfascicular nerves

#### Correlation between motor unit activities

Motor neurons innervating a muscle receive synaptic input from presynaptic neurons, supraspinal and afferent pathways. The net input to motor neurons comprises common components, that is, input to multiple motor neurons and independent ones. Although common input cannot be directly measured, it causes motor units to discharge action potentials with a degree of synchrony (correlation) and, therefore, can be inferred from the motor unit output activity by cross-correlation analysis or dimensionality-reduction methods^25,31^. Based on this methodology, prior work has shown the ability of the central nervous system to selectively trigger subsets of motor units among those innervating a muscle, irrespective of anatomical constraints^18,32–34^.

**Fig. 5:**
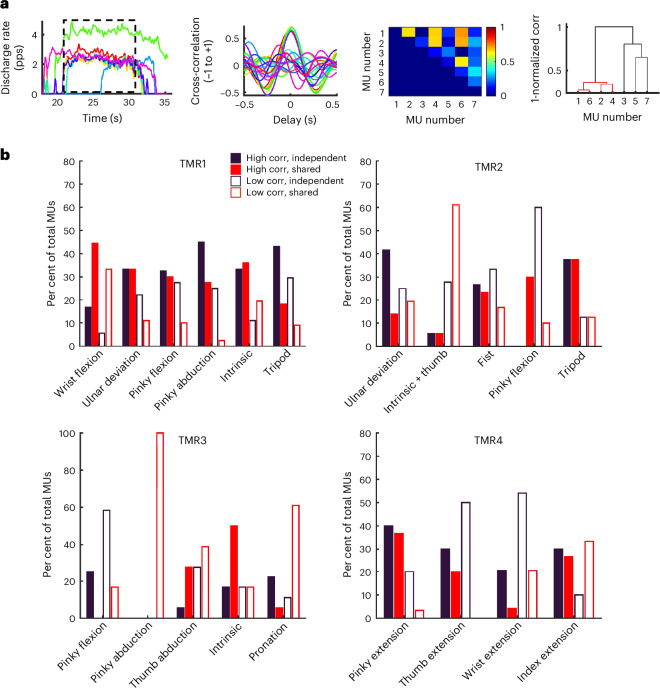
Common synaptic input analysis in MUs of reinnervated muscles. **a**, From left to right: common oscillations can be observed in the smoothed motor unit (MU) discharges (each line the activation of a MU); the presence of common synaptic input to MUs is assessed by computing the cross-correlograms between smoothed and detrended discharges of MUs (in the plateau part of the contraction); the maximum value of the cross-correlation (value between −1 and 1) within 100 ms of zero delay was used to quantify the strength of the common input. The values are reported in the cross-correlation matrix. Hierarchical clustering is applied to group MUs based on their intercorrelation. Normalized corr indicates cross-correlation values reported in the correlation matrix. corr, correlation. Following the clustering analysis, MUs receiving a higher portion of common input (correlation ≥0.5) were merged into a ‘high-correlation’ cluster (red tree branches), while a ‘low-correlation cluster’ was formed with the MUs showing a low degree of synchronization to other MUs (correlation <0.5). **b**, For each task and reinnervated muscle, we report the proportion of task-specific and shared MUs that belonged to the high- and low-correlation groups.

For each task repetition, the spike trains in the plateau phase of the reinnervated muscle contraction were smoothed with a Hanning window of 400 ms to assess the common fluctuation in the low-frequency bandwidth (<2.5 Hz); the smoothed spike trains were subsequently high-pass filtered with a cut-off frequency of 0.75 Hz (ref. ^35^). For this analysis, only tasks with more than three motor units consistently active in ≥70% of the repetitions were considered. The normalized cross-correlation function was computed between pairs of filtered spike trains and the maximum cross-correlation value within 100 ms of zero-time lag was used to quantify the strength of the common input^36^ and stored in a correlation matrix. Non-significant correlations were set to zero (‘Common synaptic input to motor units of targeted reinnervated muscles’ in Methods). Hierarchical clustering was then performed to group motor units based on their cross-correlation. The maximum correlation within each cluster was compared with a set threshold of 0.5 to merge clusters of motor units into either a high-correlated *G*_Corr≥0.5_ or a low-correlated *G*_Corr<0.5_ group. These steps are illustrated in Fig. 5a for an exemplary task. The proportion of motor units that received common input, averaged across the tasks of each reinnervated muscle, was 65.59%, 47.17%, 35.69% and 49.58% for TMR1, TMR2, TMR3 and TMR4, respectively.

Within each cluster of neurons, some neurons were exclusively associated with a single task (task specific), while others were recruited across multiple tasks (shared). In Fig. 5b, we illustrate for each task the proportion of motor units that (1) were task specific and belonged to *G*_Corr≥0.5_ (blue bar), (2) were shared and belonged to *G*_Corr≥0.5_ (red bar), (3) were task specific and belonged to *G*_Corr<0.5_ (white bar with blue edge), and (4) were shared and belonged to *G*_Corr<0.5_ (white bar with red edge). The average across-tasks percentage of task-specific motor units that received common input was 34.0% ± 10.1%, 25.8% ± 18.8%, 18.8% ± 15.4% and 31.0% ± 9.1% for TMR1, TMR2, TMR3 and TMR4, respectively. Instead, the proportion of shared motor units that received common input was 31.6% ± 8.8%, 21.4% ± 11.4%, 16.9% ± 19.5% and 18.5% ± 12.4% for the four reinnervated muscles. These results indicate that all tasks involved motor units receiving common synaptic input, with the sole exception of pinky abduction in TMR3, where no task-specific motor units could be decomposed. Moreover, common synaptic input appeared to be distributed across both task-specific and shared motor units recruited for each task. Because multiple groups of motor neurons receiving common input contributed to several tasks, the tasks were mainly performed by the coordinated (synergistic) activation of the motor neuron clusters, which is consistent with recent theories of synergistic motor neuron control^18^.

#### Neural manifolds

**Fig. 6:**
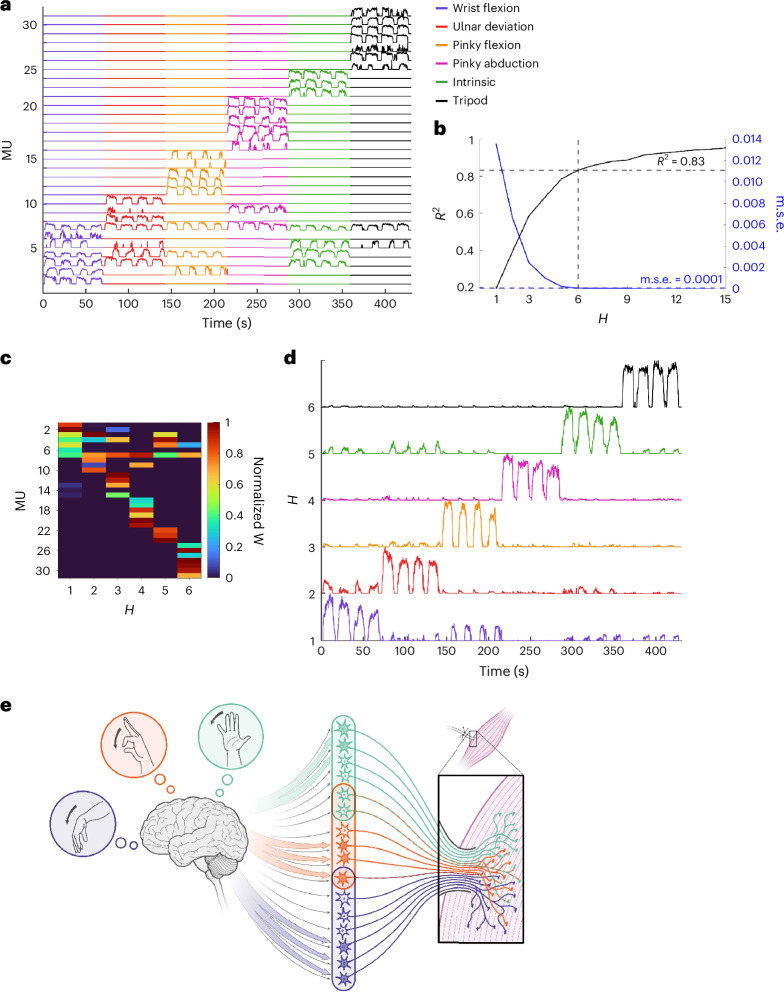
Neural manifold analysis in muscle reinnervated by a polyfunctional nerve. **a**, Smoothed motor unit (MU) spike trains from multiple tasks (colour coded) of the missing limb recorded from TMR1; MUs are tracked across tasks and spike trains are concatenated to define the MU space. **b**, An *R*^2^ curve obtained by applying NNMF to data in **a** with an increasing number of latent factors from 1 to 15 and corresponding mean squared error (m.s.e.) curve. The dashed line indicates the chosen number of latent factors (see Methods). **c**, Non-negative matrix of normalized contributions (*W*) of MUs to latent components output by NNMF **d**, Time-varying latent components *H* embedded in the MU space, extracted by NNMF. The latent signals are colour coded according to the temporal distribution of activation, given the order of recorded tasks indicated in **a**. **e**, A conceptual model of how the central nervous system encodes movement in reinnervated muscles. Different movements of the phantom limb and their corresponding cluster of MUs are represented: wrist flexion (purple), pinky flexion (orange) and pinky abduction (green). All active MUs received independent synaptic input (small grey arrows), and some received common synaptic input (big arrows). Within a cluster (that is, task), MUs may be task specific or shared among tasks (intersection between clusters). For example, there are two shared MUs for pinky flexion and abduction (orange–green) and one shared MU for pinky flexion and wrist flexion (orange–purple). Both task-specific or shared MUs may receive common input. Credit: illustration in panel **e** provided by Aron Cserveny.

In the previous section, we observed that in muscles reinnervated by polyfascicular nerves, motor units recruited for a task of the missing limb received at least a source of common synaptic input. Common inputs resulted in the observed covariation between groups of motor neuron activity. Moreover, motor neurons that receive common input may be shared across tasks. For these reasons, the high-dimensional motor unit space (that is, the space comprising the spike trains of motor units recruited across all the tasks of a reinnervated muscle) exhibits redundancy, with a dimensionality smaller than the number of active units. Therefore, we expected that the decoded motor unit activity could be modelled by a small set of latent factors, with each factor associated to a function of the missing limb. For each reinnervated muscle, we examined the lower-dimensional latent space *H* embedded in the motor unit space *X* by applying non-negative matrix factorization (NNMF)^37^. Mathematically, the problem is that of estimating two non-negative matrices *W* and *H* whose product approximates the original dataset *X*. For each reinnervated muscle, *X* is obtained by sequentially concatenating the smoothed normalized spike trains of *m* motor units active for ≥70% of repetitions *r* of all performed tasks *t*. An example of such an *m*-dimensional motor unit space *X* is shown in Fig. 6a: the concatenated neural recordings are plotted, with temporal segments colour coded to indicate the different tasks. *W* (Fig. 6c) is the non-negative basis matrix of dimension *m* (determined by total number of unique motor units measured across tasks) by *l* (dimension of the latent space), and *H* (Fig. 6d) contains the *l* non-negative time-dependent latent variables. Each column of *W*^*i*^ represents the contribution of the *m* motor units to the latent signals *l*. NNMF requires the definition of the number of latent factors a priori.

**Extended Data Fig. 1:**
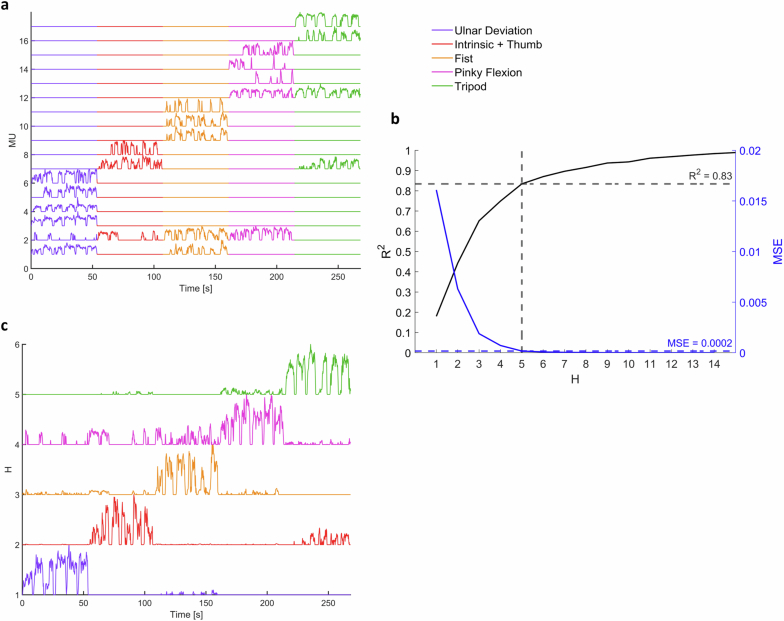
Neural manifold analysis in TMR2 muscle reinnervated by a polyfascicular nerve. **a**, Smoothed motor unit (MU) spike trains decomposed from intramuscular recordings in TMR2 for multiple tasks (color-coded) of the missing limb; we investigate the latent space embedded in the space defined by these motor units using Non-Negative Matrix Factorisation (NNMF). **b**, *R*^2^-curve obtained by applying NNMF to data in panel **a** with an increasing number of latent factors from 1 to 15 and corresponding Mean Squared Error (MSE)-curve. The dashed line indicates the chosen number of latent factors for results in panel **c**, which for TMR2 matches the number of recorded task. **c**, Time varying latent components H embedded in the motor unit space, extracted by NNMF with dimensionality obtained in **b**. Latent signals are color-coded according to the temporal distribution of activation, given the order of executed tasks indicated in **a**. Distinct patterns of activation corresponding to specific tasks are encoded by the latent space factors.

**Extended Data Fig. 2:**
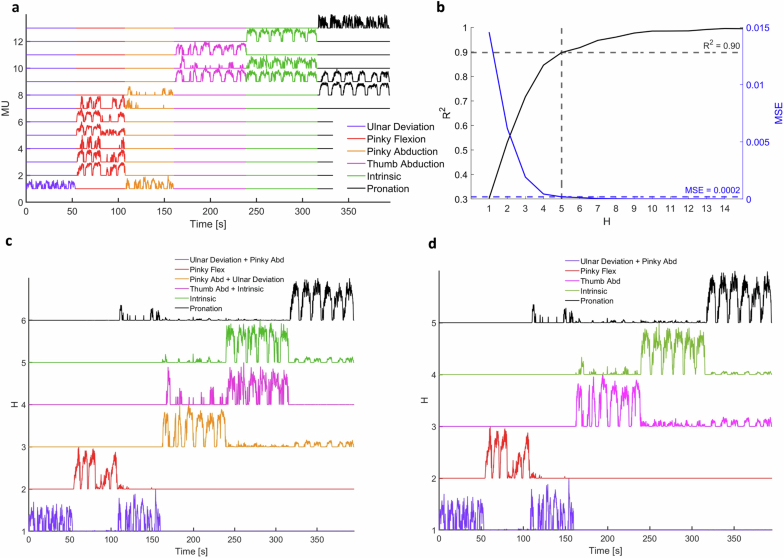
Neural manifold analysis in TMR3 muscle reinnervated by a polyfascicular nerve. **a**, Smoothed motor unit (MU) spike trains decomposed from intramuscular recordings in TMR3 for multiple tasks (colour-coded) of the missing limb; we investigate the latent space embedded in the space defined by these motor units using Non-Negative Matrix Factorisation (NNMF). **b**, *R*^2^-curve obtained by applying NNMF to data in panel (**a**) with an increasing number of latent factors from 1 to 15 and corresponding Mean Squared Error (MSE)-curve. The dashed line indicates the chosen number of latent factors for results in panel (**d**). **c**, Time-varying latent components *H* embedded in the motor unit space, extracted by NNMF with dimensionality equal to number of recorded tasks. Latent signals are color-coded according to the temporal distribution of activation, given the order of executed tasks indicated in (**a**). Distinct patterns of activation corresponding to specific tasks are encoded by the latent space factors. **d**, Time-varying latent components *H* embedded in the motor unit space, extracted by NNMF with dimensionality obtained in (b).

**Extended Data Fig. 3:**
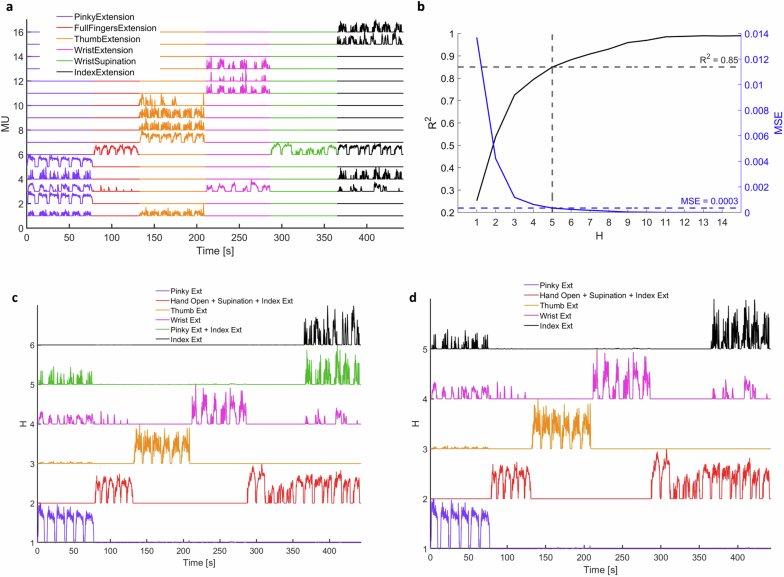
Neural manifold analysis in TMR4 muscle reinnervated by a polyfascicular nerve. **a**, Smoothed motor unit (MU) spike trains decomposed from intramuscular recordings in TMR4 for multiple tasks (colour-coded) of the missing limb; we investigate the latent space embedded in the space defined by these motor units using Non-Negative Matrix Factorisation (NNMF). **b**, *R*^2^-curve obtained by applying NNMF to data in panel (**a**) with an increasing number of latent factors from 1 to 15 and corresponding Mean Squared Error (MSE)-curve. The dashed line indicates the chosen number of latent factors for results in panel (**d**). **c**, Time-varying latent components *H* embedded in the motor unit space, extracted by NNMF with dimensionality equal to number of recorded tasks. Latent signals are color-coded according to the temporal distribution of activation, given the order of executed tasks indicated in (a). Distinct patterns of activation corresponding to specific tasks are encoded by the latent space factors. **d**, Time-varying latent components *H* embedded in the motor unit space, extracted by NNMF with dimensionality obtained in (b).

We examined the latent spaces with dimensions (1) equal to the number of measured tasks and (2) equal to the minimum number of factors beyond which an additional factor increased the coefficient of determination *R*^2^ (ref. ^38^) between the original data *X* and the reconstructed data *WH* by less than 5% (ref. ^39^). Method 2 provided a dimensionality of the latent space that matched the number of recorded tasks in TMR1 and TMR2, and a dimension of 5 was estimated for TMR3 and TMR4, which was lower than the number of tasks (six tasks were recorded for both reinnervated muscles). The values of *R*^2^ for an increasing number of latent factors are reported for all reinnervated muscles in Fig. 6b and in the Extended Data Figs. 1,b–3,b, respectively; a blue line indicated the obtained optimal number of factors.

All tasks in TMR1 and TMR2 had at least one task-specific motor unit, suggesting that the dimensionality of the neural manifold could not be lower than the number of tasks (six in TMR1 and five in TMR2). By contrast, TMR3 and TMR4 had two and one task, respectively, without task-specific motor units. In these cases, approach 2 correctly predicted that the neural signals were represented by a manifold with fewer dimensions than the total number of tasks. In Fig. 6d and Extended Data Figs. 1c–3c we provide the latent spaces of dimensions equal to the number of tasks; in Extended Data Figs. 2d and 3d, we show the latent space estimated according to the criterion 2, since it differs from the number of recorded tasks. Figure 6d (TMR1) and Extended Data Fig. 1 (TMR2) show that each dimension of the manifold had dominant activation in correspondence to a specific recorded task. Thus, the latent space dimensions effectively encoded the task-specific neural information carried by the polyfascicular nerves since at least a task-specific motor unit was active for each task. In TMR3, the manifold with dimension equal to the number of tasks with at least a task-specific motor unit (less than the total number of recorded task) allowed to separate four out of six tasks; a single latent component captured the activity of ulnar deviation and pinky abduction, which had only shared motor units. Similarly, in TMR4, the tasks with at least a task-specific motor unit could be isolated. The activity of two tasks that had only shared motor units, was captured by a single latent factor. In summary, a distinct control command could be extracted for each recorded task of reinnervated muscles 1 and 2. Distinct control commands were identified for four out of six tasks in reinnervated muscle 3 and 5 out of six tasks in reinnervated muscle 4.

We then quantified the degree of separability between each pair of time-varying activations in *H* for each reinnervated muscle using the cosine similarity measure, explained in ‘Task-dependent latent neural manifolds’ in the Methods. Low cosine similarity implies high orthogonality and, therefore, high separability of each time-varying activation. Average values of cosine similarity across tasks of each reinnervated muscle were 0.05 ± 0.03, 0.06 ± 0.02, 0.08 ± 0.08 and 0.12 ± 0.07, respectively. All tasks of TMR1 and TMR2 exhibited high separability. In fact, all tasks had at least one task-specific motor unit (independent). The higher standard deviation for TMR3 and TMR4 indicated that some tasks could not be perfectly separated. In fact, pinky flexion and ulnar deviation in TMR3, and hand open in TMR4 had no independent motor units. Cosine similarity values are reported for all reinnervated muscles in Supplementary Fig. 4.

## Discussion

TMR involves rerouting a donor nerve into a denervated muscle, allowing the muscle to receive efferent input from the donor nerve once reinnervation is complete^3^. In ref. ^40^, the concept of human–machine interface based on decoding the neural activity of reinnervated motor units was first introduced. Here, we expanded on that concept and separated the individual neural drives to reinnervated motor units for each recorded task of the missing limb using invasive microelectrode arrays^20^.

With respect to invasive solutions, surface high-density arrays allow broader muscle coverage non-invasively, with simpler deployment and higher patient acceptance, but at the cost of reduced spatial selectivity, lower bandwidth due to tissue filtering and increased variability over time due to replacement of the electrodes at each use or changes in skin conditions. Moreover, while surface arrays can support motor unit decomposition, their stability and the number of reliably trackable units remain inferior to intramuscular recordings^41^. Intramuscular recordings indeed provide higher resolution, signal fidelity and access to deep muscle fibres, enabling more accurate decoding of motor intent^42^. High-density multichannel intramuscular microelectrode arrays, as those used here^20^, enabled the recording of multisource observation (intramuscular recording at each channel) for direct motor unit decomposition^20,41^. This allowed us to observe the behaviour of individual motor units, compute their short-term synchronization and propose a conceptual model of common input to reinnervated muscles, which also supports the neural manifold analysis findings.

We investigated four reinnervated muscles in three amputee volunteers, TMR1 and TMR2 from the first and second participant and TMR3 and TMR4 from the third participant (Table 1). The polyfascicular nerves, which previously carried complex motor commands for the now-missing upper limbs, were surgically redirected to muscles near the stump that had lost their original function^5,7^. We demonstrated that reinnervated muscles received a diverse set of neural inputs, corresponding to the multiple distinct tasks encoded by the polyfunctional nerve, making them potentially suitable for controlling a prosthetic limb with multiple degrees of freedom. The potential for decoding multiple motor commands was demonstrated by introducing and validating a biointerface on the basis of probing the reinnervated muscle connected to polyfascicular nerves, with microelectrode array technology. With this recording approach, we captured the neural activity of individual motor units as volunteers performed various tasks with their missing limbs. Even though we sampled intramuscular EMG activity from each reinnervated muscle at a single, small physical site (with recording channels distributed along just 2 cm), the high resolution provided by motor unit spike detection^40^ enabled us to differentiate neural signals corresponding to distinct tasks. These tasks ranged from fine individual finger movements to more complex grasping actions, demonstrating the biointerface’s ability to decode multiple, heterogeneous neural commands from a single muscle.

Considering 23 tasks recorded across the four reinnervated muscles, 111 motor units were reliably decomposed from intramuscular EMG signals. The firing properties of these reinnervated motor units, such as the mean firing rate and CoV, were within physiological ranges, as reported for physiologically innervated muscles. In TMR3 and TMR4, the higher CoV may be due to the quality of the provided visual feedback during task execution, as well as the participant’s ability to perform the task and maintain a stable contraction. These results are in agreement with those previously reported by Kapelner et al.^43^, where no statistically significant difference in mean ISIs and CoV was observed in reinnervated motor units and physiologically innervated motor units.

We assessed the common synaptic input to motor neurons rerouted into a targeted muscle following TMR in human participants. Correlation analysis of motor unit spike trains during task-specific recruitment revealed highly correlated units, indicative of shared synaptic inputs. In addition, both task-specific and shared motor units were found to receive common synaptic inputs. These results substantially expand our understanding of the neural control of a missing limb with respect to previous studies in TMR participants that used high-density surface EMG^40^ and in animal models^20^. Each task was performed with the activation of one or more clusters of motor units. However, in 19 of the 23 tasks, at least one task-specific motor unit was uniquely recruited. These findings demonstrated that reinnervation via polyvalent nerves led to motor units that were spatially distributed in the muscle, with muscle fibres belonging to different motor units intermingled with each other (‘Motor unit activity’). However, despite the absence of spatial clustering of motor units recruited for the different tasks, motor unit groups could be defined on the basis of their task/functional role.

The covariation between motor units recruited for a task, and the presence of shared motor units between tasks indicated that the underlying neural activity was confined to a neural manifold of dimension lower than the number of identified motor units. We identified the neural manifold underlying the pooled data and, hence, the structure of the common inputs to motor neurons determining the neural activity for the different tasks, using NNMF. In TMR1 and TMR2, most of the variance within the data obtained from concatenating motor units recruited for six and five tasks, respectively, was explained by six and five latent components. Thus, each dimension of the manifold constituted a control signal for a particular task. For TMR3 and TMR4, we concatenated motor units recruited during six tasks for each reinnervated muscle. In both cases, the variability in the data was explained by a five-dimensional manifold, which agreed with our preliminary analysis, indicating that two tasks of TMR3 and TMR4 had only shared motor units. These tasks had only few motor units decomposed either because the neural activity for the tasks was not in the pick-up range of the microelectrode array, or the participants were not able to execute such tasks. Future work may further analyse the limitations imposed by sampling a limited number of motor units and the impact on motor unit-based control of a virtual or real prosthesis. Moreover, semisupervised NNMF may be explored to capture less dominant patterns in motor unit activity and maximize the number of latent signals associated with tasks of the missing limb that can be extracted from intramuscular recordings. One could argue that motor units classified as task specific may not have been observed due to fluctuations in the drive to motor units. However, the reported observations were consistent across multiple repetitions of each task and for multiple motor units. Moreover, while there is no means of assessing if the participants were truly formulating the motor intent about a task, we observed consistency in motor unit recruitment across repetitions of the same task.

The observed motor unit behaviour enabled us to propose a conceptual model illustrating how the central nervous system encodes movements post reinnervation (Fig. 6e). According to this model, each task is defined by a specific set of motor units, with some being uniquely recruited for a particular task (task-specific) and others being activated across multiple tasks (task-independent or shared). Both task-specific and shared motor units may receive common synaptic input, which synchronizes their activities. This pattern mirrors the control mechanisms observed in able-bodied individuals, where the central nervous system distributes common input across motor neurons, promoting modularity and reducing the complexity of control through dimensionality reduction^18^. This modularity was preserved in our experimental conditions, following TMR. Indeed, motor neurons did not behave independently but were grouped in clusters of common activity. Therefore, the fundamental strategy of the central nervous system of activating motor neurons with a reduced number of common inputs was maintained following TMR. This strategy could be identified by the analysis of the manifold underlying the motor unit activity.

The findings of this study offer strong evidence of high-information transfer through TMR with polyfascicular nerves, overturning previous assumptions about the limitations of EMG in decoding information from reinnervated muscles. One such limitation is the perceived lack of isolation and specificity in control signals, stemming from the reinnervation of a single muscle by multiple nerve fascicles. Another concern is the incomplete representation of nerve functions at the innervation site, especially when the transferred nerve is at a level where fascicle organization may be disrupted, leading to an unequal distribution of encoded functions. Our results suggest that these limitations may be fully overcome when employing advanced recording and decoding techniques.

RPNIs have been proposed as a solution to overcome the aforementioned limitations. However, RPNIs face challenges. Although previous research has shown that RPNI surgery can create functionally selective units^11^, there are inherent physical limitations to how many RPNIs can be generated from a single donor nerve. Furthermore, reliable techniques for extracting stable and usable EMG signals from the RPNI units for effective prosthetic control have not yet been fully established, leaving some key problems unresolved.

This study showcases the use of polyfascicular nerve transfers, combined with high-density selective recordings and advanced decoding techniques, to establish a highly efficient biointerface with the potential to control prosthetic limbs with greater functional precision and specificity. We demonstrated that diverse tasks associated with the missing limb can be effectively decoded using a single high-density microelectrode array. We conducted an offline analysis, eliminating factors that could interfere with the analysis, such as during real-time prosthetic control. Crucially, the principles outlined here are extendable to cases where multiple polyfascicular nerves are transferred to a single muscle, as shown in preclinical work by Luft et al.^44^. Our long-term objective is to create a hyper-reinnervated muscle, capable of replicating the full neural activity of the missing limb by integrating inputs from multiple polyfascicular nerves. This study supports prior findings in animal models^20^, confirming the feasibility of polyfascicular nerve transfers for sophisticated prosthetic control. However, as opposed to surface electrodes, invasive systems introduce technical complexity, especially for a possible chronic implant application, and potential risks associated with longevity of implants (for example, encapsulation, migration or device failure). Nevertheless, this approach opens new possibilities for more advanced prosthetic solutions in the future, providing a foundation for enhanced motor control and functional specificity. Future developments aiming to minimize the invasiveness of implanted systems while maximizing coverage and longevity could mitigate technical complexity, simplify the deployment and increase patient acceptance.

## Methods

This study was approved by the Ethical Committee of Imperial College London (reference number 19IC5641) and performed according to the Declaration of Helsinki. Three male participants (P1, P2 and P3) who suffered from an amputation of their upper extremity and also underwent TMR surgery were included in this study. Each volunteer provided written informed consent and a physical examination of their reinnervated muscles was performed before participation. P1 underwent his TMR surgery at the Royal Free Hospital (7 years prior) and P2 (8 years prior) and P3 (12 years prior) at the Medical University of Vienna. For P1, the ulnar nerve was transferred to the pectoralis minor muscle, and the median nerve was transferred to the lower (abdominal) part of the pectoralis major muscle. To make the EMG signals from the pectoralis minor easier to detect, the muscle was released from the coracoid process and was transferred into the axilla. Standard TMR procedures for a transhumeral amputation (P2) and a glenohumeral amputation (P3) were performed^6,14^. In P2, the ulnar nerve was transferred to the short head of the biceps, the median nerve to the brachialis and the split deep radial branch to the lateral head of the triceps and brachioradialis. For P3, the musculocutaneous nerve was transferred to the clavicular part of the pectoralis major, the ulnar nerve to the pectoralis minor, the median nerve to the sternocostal and abdominal part of the pectoralis major and the radial nerve to the latissimus dorsi and teres major. The procedure for the nerve-to-nerve coaptation in TMR surgery, where the donor nerve is sutured to the recipient nerve, is described by Pettersen et al.^45^. The stitches are positioned centrally in the donor’s nerve and further sutures secure the epineurium (donor) to the fascia and epimysium (recipient). The nerve coaptation can also be performed directly at the neuromuscular entry zone. The nerve is coapted directly to the motor nerve of the muscle and its epimysium to improve stability^12^. The main indication for TMR surgery was the treatment of phantom limb pain for P1 and enhanced prosthesis control for P2 and P3. All three patients used a myoelectrical prosthesis in daily life with two (P1), four (P2) and six (P3) standard surface bipolar electrodes for prosthesis control. The patient characteristics and additional details on prosthesis control are summarized in Table 1.

A serious adverse event was reported to the Ethics and Research Governance Coordinator. The incident was resolved after a week of hospitalization, without any other complications for the participant.

### Electrophysiological recordings in targeted reinnervated muscles

The intramuscular electromyographic activity of each reinnervated muscle was measured using a microarray described by Muceli et al.^19^. The microarray included 40 platinum channels (area of 5,257 μm^2^) linearly distributed with an interelectrode distance of 500 μm over 2 cm of a double-sided polyimide structure 20 μm thick.

The insertion point for each microarray into the corresponding reinnervated muscle was identified as the most myoelectrically active part of the muscle through clinical examination (palpation, visual muscle contraction by performing different missing limb movement tasks related to the nerve transferred into the reinnervated muscle) and surface EMG measurements with the MyoBoy (Ottobock Healthcare Products GmbH). The following four reinnervated muscles were examined: for P1, the pectoralis minor innervated by the ulnar nerve (TMR1); for P2, the biceps short head innervated by the ulnar nerve (TMR2); and for P3, the pectoralis minor innervated by the ulnar nerve (TMR3) and the latissimus dorsi (TMR4) innervated by the radial nerve. After disinfecting the skin, the microarrays were inserted acutely into the muscle at a flat angle, using a hypodermic needle of a similar size to those used in conventional concentric needle recordings. After insertion, the needle was removed while ensuring the microarray stood in place in the muscle. The entire insertion procedure was aided by using a portable ultrasound scanner. The microarray was removed at the end of the experimental session. A detailed insertion procedure is reported in Supplementary Section 1. The EMG signals were recorded in monopolar configuration using a multichannel amplifier (Quattrocento, OT Bioelettronica) with a gain of 150 and band-pass filtered (10–4,400 Hz) before being sampled at 10,240 Hz using an A/D converter to 16 bit. The reference and ground electrodes were placed in areas of no significant myoelectric activity depending on the level of amputation (for example, acromion). Each microarray provided 40 active recording channels.

### Experimental protocol

Each participant sat in front of a computer screen and received visual feedback on the EMG activity recorded by the microarray in reinnervated muscle. The signal used as visual feedback was the bipolar signal derived from the microelectrode array that resulted in maximum amplitude. The volunteer was requested to perform specific movements of their missing limb while contracting the reinnervated muscle. The list of missing limb movements/tasks to include in the protocol was planned for each patient individually to account for individual needs (for example, the ability to sustain longer contractions) and according to their nerve transfer matrix. At the beginning of each task, the MVC was recorded and used to normalize the EMG signals of the contractions for the particular task. The volunteer was then requested to contract the reinnervated muscle and modulate the EMG activity to accurately track a series of target trapezoidal trajectories displayed on the computer screen. Each trapezoidal trajectory consisted of (1) a positive ramp phase where the muscle contraction had to be increased up to a percentage of MVC; (2) a constant contraction phase where the muscle contraction had to be maintained at a percentage of MVC; and finally, (3) a negative ramp down phase where the muscle contraction had to be decreased until the muscle was fully relaxed. For example, P1 had to (1) increase the muscle contraction from 0% to 10% of MVC in 4 s, (2) maintain the contraction level at 10% of MVC for 10 s and (3) decrease the muscle contraction in 4 s. The trapezoidal task was repeated four times per task. A total of 20 s of rest was allocated between each contraction to minimize fatigue for all patients.

P2 performed for each task six contractions at 10% of MVC for 5 s and decreased back within 2 s. For P3, six repetitions per task with 2 (4) s of rise, contraction at 10% (20%) of MVC for 5 s and decreased back within 2 (4) s. A total of 20 s of rest was allocated between contractions to minimize fatigue for all patients. The list of all included tasks per patient can be found in Table 1.

### Signal processing of EMG signals and quality assessment

The recorded intramuscular EMG signals were high-pass filtered at 1,000 Hz with a zero-lag first-order digital filter. The quality of the signals was assessed by computing the root mean square of 5 s of data recorded at rest before starting the trials. The channels yielding a baseline noise >15 μV were visually inspected and removed.

### Motor unit decomposition

Each channel of the microarray recorded an EMG signal given by the superimposition of the action potential propagating bidirectionally along the muscle fibres innervated by active motor neurons in the pick-up area of the electrode. Because fibres innervated by different motor neurons are intermingled, one channel might record the action potentials of multiple motor units. Moreover, the electrical activity of a motor unit might be recorded by adjacent channels depending on the position of the electrode within the motor unit territory (that is, the space defined by the fibres innervated by a motor neuron). Since each action potential is uniquely associated with a motor unit due to the high reliability of the neuromuscular junction^46^, the motor unit spike events can be extracted by explaining multiple observations of the motor unit activity using multichannel electrodes (observations). The problem of decomposition is thus formulated as a blind-source separation problem.

The EMG signals were decomposed into their constituent motor unit spike trains using the algorithm described by Holobar and Zazula^47^ and validated by Muceli et al.^19^ using the same microarrays adopted in this study. For each task, the decomposition of signals recorded by an electrode was inferred using all the data from the repetitions. The outcome of the automatic decomposition was validated by an expert investigator using EMGLAB^21^. Specifically, each EMG signal was inspected to detect decomposition errors such as missing or incorrectly assigned discharges, paying attention to instances of long or short ISIs. Detection of superimposition of motor unit potentials was aided by the multichannel recordings; each channel of the microarray may sample a different part of the motor unit territory, providing a unique observation of the motor unit electrical activity. While the action potential morphology (that is, amplitude and shape) differs across channels, the firing pattern is the same. This redundant information is used to resolve superimpositions of action potentials from multiple motor units. When the spike train of a motor unit was fully identified, EMGLAB subtracted the template of the MUAPs from the EMG signals. When the power of residual signal was comparable with the baseline noise level the decomposition was considered completed. Small potentials and potentials resulting in bursts of activation were not decomposed for lack of accuracy. This procedure was repeated for each of the 40 channels of the electrode. To ensure that the same motor unit was being identified throughout the repetitions, we calculated the average MUAP per repetition by spike-triggered averaging, that is, by averaging the EMG signal of each channel on the intervals of 20 ms centred around the motor unit discharges obtained from decomposition of a repetition. We then computed the coefficient of determination *ρ* between the normalized MUAP templates and the ones of the other repetitions; in addition, motor units were the same across repetitions if their action potentials had maximum peak-to-peak amplitude on the same channel. Finally, we identified motor units that were satellite potentials of other units by computing the rate of agreement^48^ between pairs of discharge patterns. The rate of agreement was defined as the ratio between the number of discharges present in both discharge patterns (common) and the sum of the number of common discharges and the number of discharges present in only one of the two discharge patterns. A tolerance of ten samples (<1 ms) was used when identifying common discharges to account for propagation delays between the main potential and the satellite one. If the rate of agreement between two motor units exceeded 80%, the motor unit with later discharges was considered a satellite potential of the first motor unit, removed from the motor unit list.

As a result of automatic and manual decomposition, the firing instances of the active motor units on each of the 40 channels were obtained.

### Screening of motor units

We defined the following inclusion criteria to retain identified motor units for further analysis: (1) motor units with few sparse firings were removed; and (2) for a given task, we excluded those motor units that did not fire in at least 70% of the task repetitions and had less than 30 firings. We assessed that removing a fraction of motor units did not bias the common input and neural manifold analysis by repeating such analysis without prior removal of motor units active in less than 70% of repetitions. Given the impossibility of assessing whether participants were performing a particular task, we relied on the consistency of motor units’ recruitment across repetitions to retain or discard motor units. A value of 70% allowed to retain a reasonable number of repetitions for different participants (three out of four reps in P1 and four out of six reps in P2 and P3). (3) We consider only motor units whose spike trains were identified with a pulse-to-noise ratio ≥30 dB (ref. ^26^), a signal-to-interference metric introduced to quantify how accurately a motor unit signal can be distinguished given the signal background noise.

### Firing properties of motor units

The following analysis was performed for each repetition of the different tasks. The histogram of the ISIs for each active motor unit was computed using 1-ms bins. ISIs longer than 250 ms were considered as reflecting pauses in motor unit tonic activity and were removed from the ISIs of the motor unit. The distribution of the ISIs was tested for normality using the D’Agostino–Pearson test (*α* = 0.95)^28^ before characterizing the average firing rate of motor units. The MFR was computed as the median of the ISIs of motor units, as these had a non-normal distribution. The variability of the motor unit discharges was quantified using the CoV (in percentage), computed as the ratio between the median and the standard deviation of ISIs. The smoothed firing rate for a motor unit was calculated by passing a Hanning window of 0.4 s over the impulse train corresponding to the firing times of that motor unit (instantaneous firing rate). The quantitative values of the properties below are reported in Supplementary Table 1.

### Morphological properties of motor units

The territory of a motor unit is defined as the subarea of the cross-sectional area that encloses all the fibres belonging to a single motor unit. While a direct measurement of the motor unit territory is not possible in vivo, we estimate the territory by considering the distribution of the electrical potential across the intramuscular channels. The MUAP template, centred in a 20-ms window, is stored across the channels to build an image of the spatial distribution of the potential. We thus obtain an image where the pixel intensity corresponds to the potential amplitude. The absolute value is taken. The area occupied by the MUAP is segmented using a threshold of 15% of the maximum absolute amplitude. The number of pixels occupied by the MUAP is normalized by the total number of pixels in the two-dimensional image. Given the MUAP area, we estimate the duration (in milliseconds) of the MUAP and the MUAP cross-section as the maximum duration across the channels obtained by projecting the area on the time axis and the cross-section as the number of channels spanned by the MUAP. The peak-to-peak unipolar amplitude (in microvolts) is computed on the channels where the MUAP has maximum value. The MUAP size (μV × ms) is computed as the product between the peak-to-peak unipolar amplitude and the duration of the MUAP. For each parameter, the mean and standard deviation are reported.

### Motor unit tracking across tasks

Given the list of motor units decomposed for a task of a reinnervated muscle, we assess whether those units were recruited for other tasks of the same reinnervated muscle (that is, motor units shared across tasks). For each pair of motor units of two tasks we calculated, the (1) coefficient of determination between the normalized MUAPs template obtained by spike-triggered averaging in a 20-ms window on the channel where the peak-to-peak unipolar amplitude of MUAPs was the largest; and (2) the distribution of the MUAP across channels was estimated as detailed above by concatenating the average MUAPs obtained for each channel to build an image of the spatial distribution of the MUAP potential (channels × time × motor unit amplitude). A 15% threshold was applied to the absolute amplitude of pixels and used to segment the area occupied by the MUAP. The MUAP cross-section (that is, channels spanned by MUAP) was obtained by projecting the segmented area on the channel axis. Two MUAPs were flagged as belonging to the same motor units if *ρ* ≥ 0.85 or if their cross-section overlapped, and visual inspection assessed the match between two motor units.

### Common synaptic input to motor units of targeted reinnervated muscles

Motor unit synchronization was estimated with a commonly employed method^49^ based on the computation of the cross-correlograms between pairs of motor unit spike trains. For each repetition of each movement, we computed the cross-correlograms between all pairs of low-pass filtered motor unit spike trains considering discharges in the constant force phase of the contraction. The spike trains were smoothed using a Hanning window of 0.4 s to retain the low-frequency oscillations in the signals (<2.5 Hz, effective drive^31^) and limiting the effect of the nonlinear relationship between synaptic input and output signal^50^. The smoothed spike trains were then high-pass filtered with a cut-off frequency of 0.75 Hz to remove the offset and trend^35^. The peak of the normalized cross-correlation function within 100 ms lag is considered as an index of the common drive^36^ and used to create a correlation matrix. As a result, each task repetition had an associated cross-correlation matrix describing the synchronization between active motor units. The statistical significance of each cross-correlogram (hence of each element in the cross-correlation matrices) was assessed as in Hug et al.^18^. Each task had a correlation matrix and a corresponding significance matrix used to set to zero non-significant correlations. Hierarchical clustering was performed to group motor units of a task according to their intercorrelation. A cut-off value of 0.5 was used to merge clusters of motor units into two clusters of high (≥0.5) and low (<0.5) correlated motor units. This value was consistent across reinnervated muscles and coherent with the one obtained automatically when imposing the number of clusters equal to two.

### Task-dependent latent neural manifolds

For each reinnervated muscle *i*, the neural space embedded in the space defined by motor units decomposed for multiple tasks *X*^*i*^ is estimated using NNMF. The matrix *X*^*i*^, of dimension *m* by (*r* × *t*), is obtained by (1) concatenating the smoothed spike trains of motor units active for ≥70% of repetitions *r* of all performed tasks *t* and (2) by normalizing the smoothed spike trains to have unit variance. The variable *m* indicates the total number of independent motor units across tasks; hence, smoothed spike trains of motor units shared across tasks are located in the same row of *X*^*i*^. According to NNMF, *X*^*i*^ is mathematically described as follows:

1 $${{{X}}}^{\;i}\approx {{{W}}}^{\;i}{{{H}}}^{i},$$

where *W*^*i*^ is the non-negative basis matrix *X*^*i*^ of dimension *m* by *l* and *H*^*i*^ of dimension *l* by (*r* × *t*) is the non-negative matrix of latent time-dependent variables. Each column of *W*^*i*^ represents the contribution of the *m* motor units to the latent signals *l*. Each row of *H*^*i*^ is a latent variable comprising the time-dependent input to motor units. NNMF solves a non-convex optimization problem (that is, minimizes the reconstruction error defined as the Euclidean distance between *X*^*i*^ and *W*^*i*^*H*^*i*^), prone to converge to local minima. For this reason, given *l*, NNMF is repeated ten times with random initialization of *W*^*i*^ and *H*^*i*^ (ref. ^51^), and random shuffling of motor units spike trains in *X*_*i*_ and task repetitions concatenation order; the maximum number of iterations is set to 100. Two latent spaces are explored by (1) imposing *l* equal to the number of tasks *t* and by (2) setting *l* equal to the minimum number of latent variables explaining a significant portion of the total variance within *X*^*i*^. For the latter, various methods have been proposed: d’Avella et al.^52^ and Clark et al.^39^ selected *l* as the minimum number of latent variables beyond which an additional one increased the variance accounted by the latent variables less than 5%. The variance accounted for was computed as $1-\frac{{\mathrm{s.s.e.}}}{{\mathrm{s.s.t.}}}$, where the sum of squared errors (s.s.e.) was the unexplained variance, and the total sum of squares (s.s.t.) was the total variance (of the data). The authors of ref. ^53^ computed the mean squared error (m.s.e.) curve by linearly fitting the *R*^2^-curve with an increasing number of latent variables; the optimal number of latent variables corresponded to the maximum number of latent variables for which the m.s.e. remained lower than 10^−5^. A threshold value 5 × 10^−4^ was set in ref. ^54^. In our study, NNMF is applied for values of *l* ranging from 1 to 15. The coefficient of determination *R*^2^ is computed as in ref. ^38^. The m.s.e. values are reported for completeness. The number of latent variables is determined using the *R*^2^-curve as in ref. ^52^ and ref. ^39^.

The similarity between pairs of latent variables within each neural manifold (proxy of separability between neural drives to tasks of a reinnervated muscle) was quantified using the cosine similarity measure. The cosine similarity is defined as the pairwise dot product, divided by the norm of each latent variable. This metric was chosen because invariant to scaling of latent variables but sensitive to other linear transformations such as shifts.

### Statistical analysis and reproducibility

Shapiro–Wilk tests were used to confirm homogeneity of variance and normal distribution of data, respectively. Kruskal–Wallis one-way analysis of variance analysis was undertaken, as data violated parametric assumptions and used to analyse differences between reinnervated muscles for specific properties of motor units. The D’Agostino–Pearson test was used to test the ISIs distribution for normality. Statistical significance was assumed at *P* ≤ 0.05. All data are reported as the mean ± standard deviation.

The following steps were implemented to ensure reproducibility: (1) the analysis reported in the ‘Results’ uses only motor units that could be accurately decomposed and were detected in ≥70% of the repetitions of the particular task; (2) we used an automatic EMG decomposition algorithm extensively validated on various datasets and manually assessed the results using the spike-sorting software EMGLAB to identify and correct decomposition errors; and (3) tracking of motor units was done automatically and visually inspected by an expert examiner to ensure correctness.

### Reporting summary

Further information on research design is available in the Nature Portfolio Reporting Summary linked to this article.

## Supplementary information

Supplementary Information Supplementary Fig. 1 in discussion session and Figs. 2–4.

Reporting Summary

Peer Review File

## Data Availability

The data supporting the findings of this article are included in the Article and its Supplementary Information. Raw data may be available from the corresponding authors upon reasonable request. Restricted access to the data is intended to protect participants’ identities, as the limited number of patients undergoing TMR and the few clinics performing the procedure increase the risk of reidentification.

## Code availability

The code for figure generation and the analysis is available from the corresponding authors upon reasonable request.
