# Author Correction: Implanted microelectrode arrays in reinnervated muscles allow separation of neural drives from transferred polyfunctional nerves

**DOI:** 10.1038/s41551-026-01711-w

**Published:** 2026-05-20

**Authors:** Laura Ferrante, Anna Boesendorfer, Deren Y. Barsakcioglu, Benedikt Baumgartner, Yazan Al-Ajam, Alexander Woollard, Norbert V. Kang, Oskar C. Aszmann, Dario Farina

**Affiliations:** 1 Department of Bioengineering, Imperial College London, London, UK; 2 Clinical Laboratory for Bionic Extremity Reconstruction, Department of Plastic, Reconstructive and Aesthetic Surgery, Medical University of Vienna, Vienna, Austria; 3 Plastic Surgery Department, Royal Free Hospital NHS Trust, London, UK; 4 Department of Plastic, Reconstructive and Aesthetic Surgery, Medical University of Vienna, Vienna, Austria

**Keywords:** Computational neuroscience, Motor neuron, Machine learning, Biomedical engineering, Microarrays

Correction to: *Nature Biomedical Engineering* 10.1038/s41551-025-01537-y, published online 24 October 2025.

In the version of this article initially published, there was a typographical error in the first paragraph of the “Task-dependent latent neural manifolds” section of the Methods, where in the text now reading “The authors of ref. 53 computed the mean squared error (m.s.e.) curve by linearly fitting the *R*^2^-curve with an increasing number of latent variables; the optimal number of latent variables corresponded to the maximum number of latent variables for which the m.s.e. remained lower than 10^−5^,” “10^−5^” was shown as “10^5^.” The text is amended in the HTML and PDF versions of the article.

