# Supplementary material for: Implanted microelectrode arrays in reinnervated muscles allow separation of neural drives from transferred polyfunctional nerves
Source: Nat Biomed Eng. 2025 Oct 24;10(7):1485–500. doi: 10.1038/s41551-025-01537-y (PMC13375542; doi:10.1038/s41551-025-01537-y)
Supplement: Supplementary file 1 — Supplementary Fig. 1 in discussion session and Figs. 2–4. [file 41551_2025_1537_MOESM1_ESM.pdf]

nature biomedical engineering

Supplementary information

https://doi.org/10.1038/s41551-025-01537-y

Implanted microelectrode arrays in reinnervated muscles allow separation of neural drives from transferred polyfunctional nerves

In the format provided by the authors and unedited

Contents

1 Procedure for micro-electrode array insertion **2**

2 Properties of motor units **2**

3 Satellite potentials **3**

4 Motor unit tracking and signal stability **4**

5 Cosine similarity as a measure of neural drive separability **5**

1 Procedure for micro-electrode array insertion

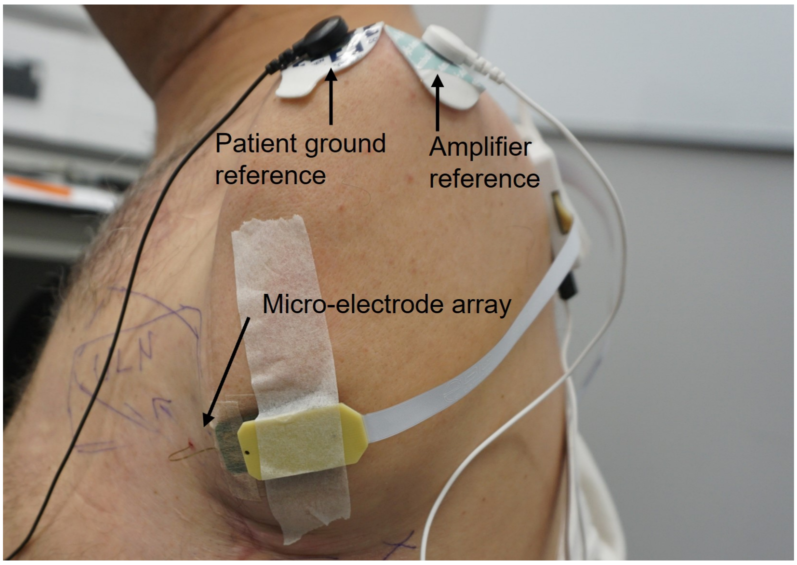

Supplementary Information Figure 1 Example of experimental setup for participant P3. The micro-electrode was inserted percutaneously in the pectoralis minor reinnervated by the ulnar nerve (TMR3). The ground and amplifier reference electrodes were placed at the acromion.

The skin of the insertion area was disinfected before the micro-electrode array was implanted following these steps while aided by a portable ultrasound probe: (1) a hole was made with a hypodermic needle to break the skin and adipose layer barrier for smoother insertion of the micro-electrode array; (2) the package containing sterilized micro-electrode array was opened and checked to ensure none of the components were damaged; (3) the EMG needle was inserted with a flat angle (approximately 45°) through the skin into the muscle (max 2.5 to 3 cm); (4) the needle was kept inside while cutting the glue of the tiny filament; (5) the needle was removed leaving only the wire with the EMG channels inside the muscle. The electrodes were then fixed with biocompatible tapes; (6) the participant and amplifier references were placed on the acromion for P3 or with a wet textile band on the upper arm for other participants.

2 Properties of motor units

Task	**MFR** [Hz]	**CoV** [%]	**Amplitude** [µV]	**Duration** [ms]	**MUAP size** [µV x ms]	**Normalised MUAP area**
**TMR1**						
Wrist Flexion	11.95 ± 4.03	0.20 ± 0.07	52.97 ± 44.96	1.97 ± 0.52	96.50 ± 72.79	0.01 ± 0.01
Ulnar Deviation	13.26 ± 3.61	0.21 ± 0.07	27.63 ± 24.40	2.08 ± 0.51	47.25 ± 26.58	0.03 ± 0.03
Pinky Flexion	14.33 ± 4.47	0.27 ± 0.09	48.04 ± 42.01	2.00 ± 0.63	87.99 ± 90.71	0.02 ± 0.04
Pinky Abduction	13.84 ± 3.68	0.22 ± 0.11	41.57 ± 23.08	2.07 ± 0.62	79.29 ± 44.67	0.02 ± 0.03
Intrinsic	11.24 ± 3.69	0.19 ± 0.05	48.98 ± 52.52	2.29 ± 0.86	92.65 ± 102.06	0.03 ± 0.04
Tripod	11.19 ± 2.38	0.28 ± 0.08	15.94 ± 8.99	2.51 ± 0.76	35.33 ± 16.48	0.02 ± 0.01
**Mean ± std**	**12.43 ± 3.46**	**0.23 ± 0.06**	**39.88 ± 39.24**	**2.18 ± 0.63**	**75.40 ± 73.06**	**0.02 ± 0.03**
**TMR2**						
Ulnar Deviation	12.68 ± 3.96	0.28 ± 0.11	145.09 ± 102.40	1.22 ± 0.88	119.28 ± 67.55	0.01 ± 0.01
Thumb (+ Intrinsic)	14.16 ± 3.92	0.33 ± 0.12	183.92 ± 101.53	2.03 ± 0.50	388.38 ± 281.40	0.01 ± 0.01
Flexion of Fingers	15.15 ± 3.03	0.38 ± 0.12	171.32 ± 94.21	1.54 ± 0.94	199.69 ± 60.89	0.01 ± 0.01
Pinky Flexion	13.18 ± 4.16	0.36 ± 0.12	51.82 ± 30.76	1.58 ± 0.45	74.14 ± 30.50	0.01 ± 0.01
Tripod	11.43 ± 1.91	0.27 ± 0.07	80.94 ± 54.33	2.58 ± 2.21	112.37 ± 67.10	0.03 ± 0.04
**Mean ± std**	**13.35 ± 2.89**	**0.32 ± 0.06**	**129.36 ± 91.36**	**1.72 ± 1.19**	**168.02 ± 149.62**	**0.01 ± 0.02**
**TMR3**						
Pronation	17.42 ± 2.88	0.43 ± 0.20	127.35 ± 59.02	1.49 ± 0.67	221.55 ± 184.60	0.01 ± 0.01
Ulnar Deviation	10.63 ± 2.30	0.37 ± 0.09	18.93 ± 13.04	3.87 ± 1.043	76.28 ± 57.47	0.03 ± 0.02
Pinky Flexion	14.07 ± 3.79	0.30 ± 0.11	73.42 ± 39.04	2.11 ± 0.90	144.38 ± 88.25	0.01 ± 0.01
Pinky Abduction	13.52 ± 2.15	0.35 ± 0.06	16.12 ± 1.79	4.99 ± 0.39	80.52 ± 10.51	0.04 ± 0.01
Thumb Abduction	20.15 ± 4.39	0.37 ± 0.11	88.97 ± 15.60	1.11 ± 0.47	94.27 ± 32.05	0.01 ± 0.00
Intrinsic	15.85 ± 4.39	0.39 ± 0.11	135.28 ± 118.87	2.43 ± 1.98	205.28 ± 201.44	0.02 ± 0.02
**Mean ± std**	**15.33 ± 3.76**	**0.36 ± 0.09**	**83.76 ± 73.59**	**2.32 ± 1.47**	**144.57 ± 137.99**	**0.01 ± 0.01**
**TMR4**						
Wrist Extension	17.83 ± 1.41	0.54 ± 0.10	91.45 ± 20.06	2.06 ± 0.65	178.77 ± 31.97	0.04 ± 0.02
Supination	19.54 ± 1.13	0.21 ± 0.00	32.89 ± 0.25	2.84 ± 0.14	93.42 ± 3.85	0.03 ± 0.00
Index Extension	16.49 ± 2.49	0.38 ± 0.18	85.09 ± 46.39	2.41 ± 0.49	187.14 ± 68.44	0.03 ± 0.01
Pinky Extension	21.16 ± 4.35	0.47 ± 0.27	66.64 ± 47.83	2.39 ± 0.87	146.34 ± 83.24	0.03 ± 0.01
Thumb Extension	20.39 ± 4.14	0.47 ± 0.23	59.03 ± 50.21	2.92 ± 0.81	147.93 ± 95.72	0.04 ± 0.02
Extension of Fingers	14.65 ± 1.38	0.21 ± 0.07	35.91 ± 2.31	3.07 ± 0.25	110.24 ± 9.13	0.03 ± 0.01
**Mean ± std**	**19.13 ± 3.44**	**0.44 ± 0.17**	**71.79 ± 45.92**	**2.54 ± 0.66**	**160.84 ± 77.65**	**0.03 ± 0.01**

Supplementary Information Table 1 Average value of motor unit properties across task repetitions for all reinnervated muscles TMR1-4. Median firing rate [Hz], Peak-to-peak unipolar amplitude [µV] computed on the channel where the MUAP had maximum amplitude, duration of MUAP [ms] computed on the channel where the MUAP had maximum duration, normalized area occupied by the MU potential across the 40 channels considering a 20 ms time window centred at the MUAP main peak. A detailed description of such properties is provided in the Methodology section of the manuscript.

3 Satellite potentials

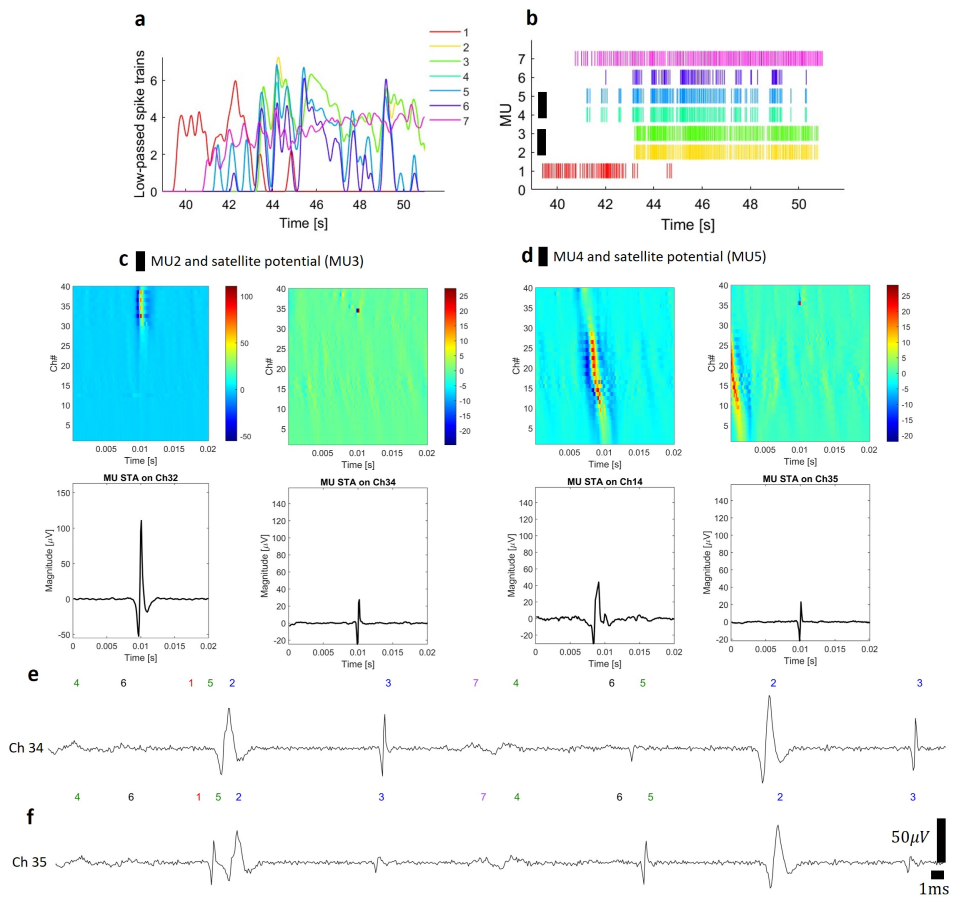

Supplementary Information Figure 2 Example of satellite potentials observed in intramuscular recordings from reinnervated muscles. **a,** Seven motor units are decomposed during trial 4 of Index Extension in TMR4. **b,** The smoothed spike trains obtained by low-pass filtering the instantaneous discharge rates of individual motor units are shown. Visual inspection of the signals reveals that the spike trains of motor units 2 and 3, and MUs 4 and 5 matched. The spike trigger average of the EMG signals on each channel provides the 2D-image, shown in (c) on the left and (d) on the right, of the MU potential distribution across channels of the micro-electrode array. These reveal the presence of the satellite potentials (on the right of panels c and d). **e-f,** show a portion of EMG signals recorded by channels 34 and 35 of the micro-electrode array. Annotation of detected motor units is indicated in correspondence to the instance of time the motor unit fired. The main potential and corresponding satellite potentials can be observed for MU2 and MU4.

4 Motor unit tracking and signal stability

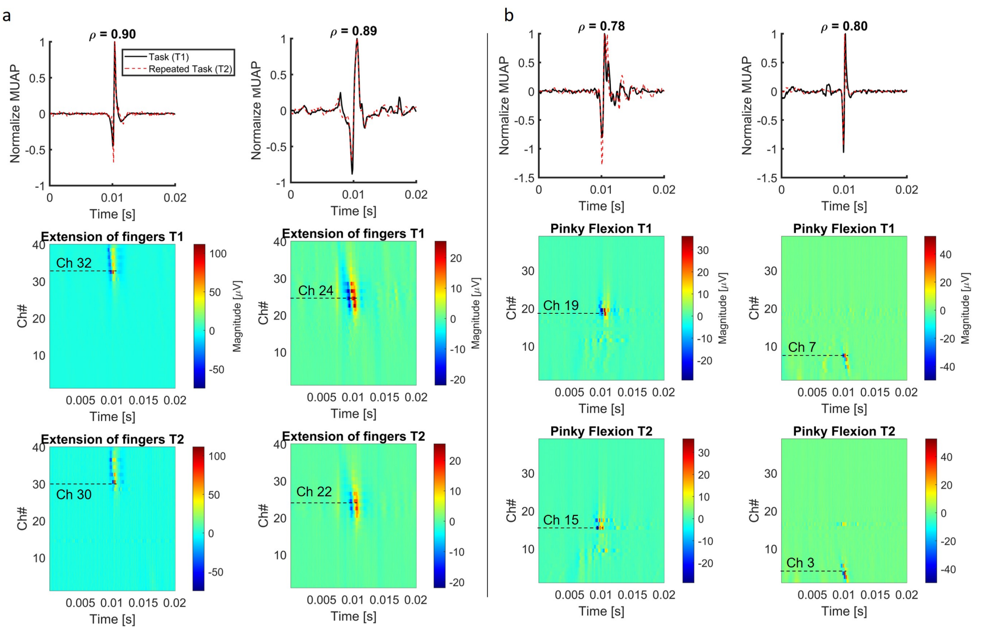

Supplementary Information Figure 3 Tracking of motor units for tasks with significant time intervals between repetitions. Additional examples of motor units detected during two instances (beginning T1 and end of experimental session T2) of Extensions of Fingers (i.e., hand opening) **(a)** and Pinky Flexion **(b)** for P3. EMG recordings at task repetitions T1 and T2 were decomposed separately to identify motor units. The normalised MUAP of the matched motor units is shown (black and red dotted line) and the goodness of the fit between the two is quantified by the coefficient of determination (top figures in panel a ns b). The remaining figures in the two panels show the average distribution of motor unit action potential during T1 and T2, respectively. The dotted lines indicate the channel (Ch) at which the average MUAPs had maximum peak-to-peak amplitude. A consistent shift of two and four channels can be observed for T1 and T2 during Fingers Extension and Pinky Flexion, respectively.

5 Cosine similarity as a measure of neural drive separability

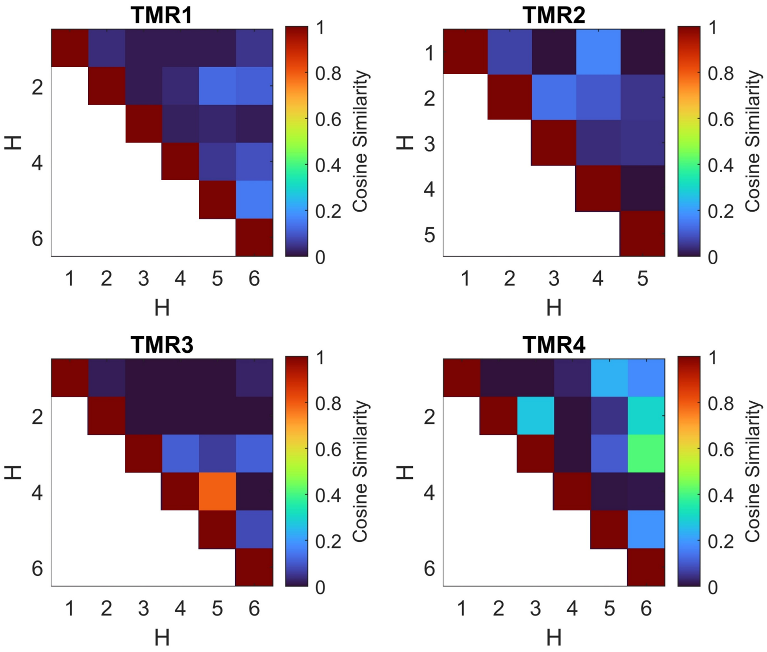

Supplementary Information Figure 4 Separability of neural drives. Cosine similarity values computed between pairs of latent factors obtained from the neural manifold analysis for each reinnervated muscle.
