# Supplementary material for: Implanted microelectrode arrays in reinnervated muscles allow separation of neural drives from transferred polyfunctional nerves
Source: Nat Biomed Eng. 2025 Oct 24;10(7):1485–500. doi: 10.1038/s41551-025-01537-y (PMC13375542; doi:10.1038/s41551-025-01537-y)
Supplement: Supplementary file 2 — Reporting Summary [file 41551_2025_1537_MOESM2_ESM.pdf]

nature portfolio

Corresponding author(s): Dario Farina, Laura Ferrante

Last updated by author(s): Jul 30, 2025

Reporting Summary

Nature Portfolio wishes to improve the reproducibility of the work that we publish. This form provides structure for consistency and transparency in reporting. For further information on Nature Portfolio policies, see our Editorial Policies and the Editorial Policy Checklist.

Statistics

For all statistical analyses, confirm that the following items are present in the figure legend, table legend, main text, or Methods section.

n/a	Confirmed	
☐	☒	The exact sample size (n) for each experimental group/condition, given as a discrete number and unit of measurement
☐	☒	A statement on whether measurements were taken from distinct samples or whether the same sample was measured repeatedly
☐	☒	The statistical test(s) used AND whether they are one- or two-sided<br>*Only common tests should be described solely by name; describe more complex techniques in the Methods section.*
☐	☒	A description of all covariates tested
☐	☒	A description of any assumptions or corrections, such as tests of normality and adjustment for multiple comparisons
☐	☒	A full description of the statistical parameters including central tendency (e.g. means) or other basic estimates (e.g. regression coefficient) AND variation (e.g. standard deviation) or associated estimates of uncertainty (e.g. confidence intervals)
☐	☒	For null hypothesis testing, the test statistic (e.g. F, t, r) with confidence intervals, effect sizes, degrees of freedom and P value noted<br>*Give P values as exact values whenever suitable.*
☒	☐	For Bayesian analysis, information on the choice of priors and Markov chain Monte Carlo settings
☒	☐	For hierarchical and complex designs, identification of the appropriate level for tests and full reporting of outcomes
☒	☐	Estimates of effect sizes (e.g. Cohen's d, Pearson's r), indicating how they were calculated

Our web collection on statistics for biologists contains articles on many of the points above.

Software and code

Policy information about availability of computer code

Data collection	Electromyographic signals were recorded using a multichannel amplifier (Quattrocento, OT Bioelettronica, Torino, Italy) and the software OTBioLab+ version 1.5.9
Data analysis	Custom software was used in MATLAB 2022b for electromyographic data decomposition. EMGLAB open-source software was used in MATLAB 2022b for editing motor unit spike trains. Custom software developed in MATLAB2022b was used for the rest of the data analysis and statistics (properties of motor units, common input analysis, neural manifold analysis). Code for generating figure and the analysis may be shared upon reasonable request.

For manuscripts utilizing custom algorithms or software that are central to the research but not yet described in published literature, software must be made available to editors and reviewers. We strongly encourage code deposition in a community repository (e.g. GitHub). See the Nature Portfolio guidelines for submitting code & software for further information.

Data

Policy information about availability of data

All manuscripts must include a data availability statement. This statement should provide the following information, where applicable:

- Accession codes, unique identifiers, or web links for publicly available datasets
- A description of any restrictions on data availability
- For clinical datasets or third party data, please ensure that the statement adheres to our policy

The data supporting the findings of this article are included in the article and Supplementary material. Raw data are available from the corresponding authors upon reasonable request.

Research involving human participants, their data, or biological material

Policy information about studies with human participants or human data. See also policy information about sex, gender (identity/presentation), and sexual orientation and race, ethnicity and racism.

Reporting on sex and gender	Gender data were not collected separately from biological sex data. Sex-related differences were not among the research hypothesis.
Reporting on race, ethnicity, or other socially relevant groupings	No groupings by race, ethnicity or socioeconomic status were performed.
Population characteristics	Three men (P1, P2, P3 aged 62, 34 and 52 at the time of data collection) who suffered from an amputation of their limb at the transhumeral (P1, P2) and glenohumeral level (P3) were included in this study. They underwent their TMR surgery at 7 to 12 years prior to the experiments, to treat neuroma and phantom limb pain (P1), and to improve prosthesis control (P2, P3). Detailed participants characteristics are reported in Tab.1.
Recruitment	Participants were recruited through emails or direct outreach.
Ethics oversight	This study was approved by the Ethical Committee of Imperial College London (reference number: 19IC5641). Informed consent was obtained from all participants before participating in the study. Participants travel, accommodation and sustainability expenses were refund. A monetary reward was provided for their participation.

Note that full information on the approval of the study protocol must also be provided in the manuscript.

Field-specific reporting

Please select the one below that is the best fit for your research. If you are not sure, read the appropriate sections before making your selection.

☒ Life sciences ☐ Behavioural & social sciences ☐ Ecological, evolutionary & environmental sciences

For a reference copy of the document with all sections, see nature.com/documents/nr-reporting-summary-flat.pdf

Life sciences study design

All studies must disclose on these points even when the disclosure is negative.

Sample size	The aim of our study was to provide a proof-of-concept of the feasibility of using polyfascicular nerve transfer and invasive multi-electrode arrays to establish a novel biointerface for prosthesis control. For this reason, a sample size of 3 participants, where 4 reinnervated muscles were analysed in total, was considered.
Data exclusions	No data were excluded from this study.
Replication	Experiments were repeated across multiple subjects. Each task in the protocol was repeated multiple times.
Randomization	No randomization was performed, as the study did not include experimental groups.
Blinding	No blinding was performed, as the study did not include experimental groups.

Reporting for specific materials, systems and methods

We require information from authors about some types of materials, experimental systems and methods used in many studies. Here, indicate whether each material, system or method listed is relevant to your study. If you are not sure if a list item applies to your research, read the appropriate section before selecting a response.

Materials & experimental systems

n/a	Involved in the study
☒	☐ Antibodies
☒	☐ Eukaryotic cell lines
☒	☐ Palaeontology and archaeology
☒	☐ Animals and other organisms
☒	☐ Clinical data
☒	☐ Dual use research of concern
☒	☐ Plants

Methods

n/a	Involved in the study
☒	☐ ChIP-seq
☒	☐ Flow cytometry
☒	☐ MRI-based neuroimaging

Plants

Seed stocks	n/a
Novel plant genotypes	n/a
Authentication	n/a
