# Supplementary material for: Implanted microelectrode arrays in reinnervated muscles allow separation of neural drives from transferred polyfunctional nerves
Source: Nat Biomed Eng. 2025 Oct 24;10(7):1485–500. doi: 10.1038/s41551-025-01537-y (PMC13375542; doi:10.1038/s41551-025-01537-y)
Supplement: Supplementary file 3 — Peer Review File [file 41551_2025_1537_MOESM3_ESM.pdf]

nature portfolio

Peer Review File

Implanted micro-electrode arrays in reinnervated muscles allow separation of neural drives from transferred polyfunctional nerves

Corresponding Author: Prof Dario Farina

Version 0:

Decision Letter:

Dear Professor Farina,

Thank you again for submitting to *Nature Biomedical Engineering* your manuscript, "Separation of Neural Drives to Muscles from Transferred Polyfunctional Nerves using Implanted Micro-electrode Arrays". The manuscript has now been seen by 3 experts, whose reports you will find at the end of this message.

You will see that the reviewers appreciate the work. However, they express concerns about the degree of support for the claims, and provide useful suggestions for improvement. We hope that with substantial further work you can address the criticisms and convince the reviewers of the merits of the study. In particular, we would expect that a revised version of the manuscript provides:

* Extended discussion on the implications and generalisation of the results showing that the lower-dimensional latent space captures key patterns related to the movement of the phantom limb, potentially with quantification.

* Extended assessment of how the chosen motor unit inclusion might have skewed the results towards already separated signals.

When you are ready to resubmit your manuscript, please upload the revised files, a point-by-point rebuttal to the comments from all reviewers, the reporting summary, and a cover letter that explains the main improvements included in the revision and responds to any points highlighted in this decision.

Please follow the following recommendations:

* Clearly highlight any amendments to the text and figures to help the reviewers and editors find and understand the changes (yet keep in mind that excessive marking can hinder readability).

* If you and your co-authors disagree with a criticism, provide the arguments to the reviewer (optionally, indicate the relevant points in the cover letter).

* If a criticism or suggestion is not addressed, please indicate so in the rebuttal to the reviewer comments and explain the reason(s).

* Consider including responses to any criticisms raised by more than one reviewer at the beginning of the rebuttal, in a section addressed to all reviewers.

* The rebuttal should include the reviewer comments in point-by-point format (please note that we provide all reviewers will the reports as they appear at the end of this message).

* Provide the rebuttal to the reviewer comments and the cover letter as separate files.

We expect that you will be able to resubmit the manuscript within 18 weeks of receiving this message. If this is the case, you will be protected against potential scooping. Otherwise, we will be happy to consider a revised manuscript as long as the significance of the work is not compromised by work published elsewhere or accepted for publication at *Nature Biomedical Engineering*.

We hope that you will find the referee reports helpful when revising the work. Please do not hesitate to contact me should you have any questions.

Best wishes,

Valeria

__
Dr Valeria Caprettini
Senior Editor, *Nature Biomedical Engineering*

____
Reviewer #1 (Report for the authors (Required)):

I started reading this paper, expecting a relatively straightforward analytical approach to maximize the information available following TMR. While this would've been similar to an earlier paper from the senior author (which they cited) the comparison to the results of those surface recordings could've been useful. However, except for the passing reference and citation, there was no such comparison made, nor any direct comparison to the information available from TMR patients by more traditional methods. The paper also contains analyses presumably meant to address basic science questions, some of them hypothesis driven, and others purely descriptive. Unfortunately, I found it rather difficult to take away any high-level conclusions from either theme. Better focus on a few central points could make this study more accessible and compelling.

Section 2.2.2 Describes the normality, mean firing rate, and CoV of the MU recordings, comparing the latter two measures across implants and to normally innervated muscle. The authors found no real patterns and did not return to these results in the discussion. It is not clear to me what they add.

Much is made of the presence of task-specific and task-independent motor units, the logic for which I find a bit difficult to follow, given that this distinction is entirely dependent on the choice of tasks. Motor units were also categorized with respect to strength of pairwise cross correlation, a measure of common input. When they compared the two measures, the authors concluded that, "common synaptic input appeared to be distributed across both task specific and shared motor units recruited for each task. This suggests that synergistic behavior between motor units is preserved even after reinnervation." It is hard for me to understand how the authors draw this final conclusion, unless they are simply arguing that some common drive remains after reinnervation, not that it is unchanged.

"We tested the hypothesis that the recorded neural activity of a reinnervated muscle could be modelled by a set of latent factors, with the number of factors being equal to or smaller than the number of measured tasks."
This is not a hypothesis, just standard dimensionality reduction.

"We then evaluated the hypothesis that each dimension of the lower-dimensional latent space (i.e., neural manifold) captured key patterns of neural activity that corresponded to a recorded movement of the phantom limb."
This is a testable hypothesis, and the result shown in Fig 6 is particularly impressive with respect to the separation between tasks. It is more or less, what I expected the paper to be about. However, this result is never quantified, and appears to be from a single nerve of a single participant. How does the information provided from this type of intramuscular recording compare to what can be obtained with high density surface electrodes? Would it be worth the added complexity? Also, unless I am missing something, NNMF, which is unsupervised (unlike dPCA), would not necessarily be expected to find a projection that separates tasks like this. This might be worth some comment in the discussion, particularly given the suggestion about the use of these signals for control. Might some further de-mixing step be necessary in some cases?

"All tasks in TMR1 and TMR2 had at least one task-specific motor unit, suggesting that the dimensionality of the neural manifold should correspond to the number of tasks."
I do not follow this logic, particularly if this is meant to describe a general concept, as opposed to a comment about this particular experimental outcome.

"Overall, a total of 19 out of the 23 recorded tasks in four reinnervated muscles could
be decoded from the proposed biointerface."
I do not understand this statement. At the very least, it must refer to classification as opposed to decoding. However, these 23 tasks were distributed across multiple nerves from multiple participants. The best this could represent is separate classifications among much smaller numbers of tasks.

"We thoroughly analyzed the functional roles of motor units consistently recorded across multiple task repetitions and observed distinct clustering based on their functional contributions…These findings demonstrate that reinnervation via polyvalent nerves leads to a functional compartmentalization within the muscle."
This seems to refer to spatial ("compartmentalization within the muscle"), as opposed to functional clustering, although I don't think anything like that was demonstrated. Did I miss it? If not, please clarify this statement.

“The covariation between motor units recruited for a task, and the presence of shared motor units between tasks indicated that the underlying neural activity was confined to a neural manifold of dimension lower than the number of identified motor units.”
Does this refer to a manifold you can compute from available data, or to a hypothetical underlying manifold? The former must be the case. The latter, clearly not, but the data can’t really address that point. Can this statement be clarified?

Specific comments

“Targeted Muscle Reinnervation (TMR) involves the transfer of polyfascicular nerves, which previously innervated muscles of the missing limb, to the motor nerves of surgically denervated target muscles [3].”
At least, as originally described in the cited publication, the anastomosis was to denervated muscle, not to a nerve.

“…as well as real-time visual feedback of the reinnervated muscle EMG activity (as recorded by the micro-electrode array),”
Some indication here, of how that was done. 1D display of a single channel?

“TMR nerve transfer surgery: a polyfascicular nerve that previously innervated multiple upper-limb muscles (dotted purple line) was transferred into a spare target muscle (continuous light purple line).”
The reference to purple lines appears to be a mistake.

“In an exemplary task repetition,”
I suspect you mean “exemplar” here and elsewhere, unless you are intentionally showing examples that are not representative of typical behavior.

“…participant P3 isometrically contracts muscle TMR4”
Why would this be considered an isometric contraction?

“…with a pulse-to-noise ratio >30 dB [24].”
Define this metric here.

“We further investigated the distribution of ISIs by computing the histogram of the inter-spike intervals with 1-ms bins (Fig.2-f): ISIs had either skewed or multimodal distributions.”
How does this and analysis differ from that of the previous paragraph? Why both?

“…median discharge rate (MFR)”
Change either the name or the abbreviation, so that they match

“Despite large variability, the MFR were within the physiological range reported for voluntary contractions of physiologically innervated muscles”
Why is this summary here, rather than in the previous paragraph where MFR was discussed?

“The mean and standard deviation values of MFR and CoV per each task of the reinnervated muscles are reported in Tab.1 of the Supplementary Material.”
Same question here. This paragraph is no longer about these values.

“…common input was 34.00°æ10.09%, 25.76°æ18.80%, 18.77°æ15.35%, and 31.04°æ9.07%”
I would suggest that you drop one or two significant figures when reporting numbers for which the standard deviation is nearly the same magnitude of the mean.

“The observed motor unit behavior enabled us to propose a conceptual model illustrating how the central nervous system encodes movements post-reinnervation (Fig.6-c).”
This is a reference to 6-e, not 6-c.

Reviewer #2 (Report for the authors (Required)):

Thanks to the authors for their submission; therein, they clearly present a novel methodology that can further the field, providing techniques to increase the fidelity and flexibility of sensors for decoding motion intention. These results could be applied to improve controllability for independent degrees of freedom in prosthetic limbs, bridging the gap towards full restoration of function.

To briefly summarize my interpretation of the work, a high-density micro electrode array was inserted into muscles of the residuum reinnervated with polyvalent neurons to study the separability of recorded EMG signals into distinct motor commands. By asking participants to ramp up, hold, and ramp down different tasks/movements, a dataset was constructed such that individual motor unit’s depolarization could be identified with clustering and dimensionality reduction techniques. It was found that both single- and multi-task motor units could be consistently extracted and the synergistic coactivation of these units allows a high variety of movements to be produced.

I have relatively few major comments, as I think the experiments performed and data were suitable for the question the

authors were answering.
1. The largest question would be the decision to exclude motor units not present in at least 70% of the tasks was reached and how that threshold was chosen. Given that the paper is an analysis of the separability of motor unit activation according to movement, could the removal of some fraction of the signals not potentially bias the results towards already-separate signals? It would be helpful to know how many total motor units were identified before trimming to the used 111; this would indicate how consistent the overall activation patterns were. Further, performing dimensionality reduction with NNMF should not have required the exclusion of those signals. As they were inconsistent, NNMF could have found a latent space of the same dimension using the (b) criteria of the paper (the coefficient of determination method) without a meaningful increase in reconstruction error. Demonstrating that the dimension of the latent space remained unchanged or increased only slightly with all motor units would be strong support justifying their exclusion.

As for minor concerns, there are a few additional comments:
1. Why were the ramping protocols varied between each participant? The most MU were found in TMR1, with P1 having the longest isometric hold. Could this have influenced the results?
2. TMR1/2 and TMR3/4 present different results in a few places (section 2.2.2 and the size of the latent spaces). The discussion section doesn’t address that these groupings occur in patients with different amputation levels, even with the same reinnervated target muscle (for TMR1/TMR3). Are there other mechanisms that could’ve impacted motor unit recruitment – like the presence of proprioceptive or sensory afferents from the residual upper arm in P1?
3. Section 4.4 describes the signal quality assessment and exclusion criteria – how many signals were used from each electrode array?
4. The text or figure captions refer to dotted lines or shaded areas in some of the figures that aren’t present – please check for consistency between the final figures and text (purple lines in figure 1, shaded blue in figure 2, blue color in figure 5)
5. A few sentences are hard to understand, especially around line 169 and line 1111. It appears that some phrases may have been combined and transition phrases were dropped

This article presents a contribution to knowledge by for the first time using source separation techniques to accomplish movement decoding in a single reinnervated muscle, as opposed to requiring surgical fascicle splitting as in rPNI construction. This presents a promising approach to increasing the number of control signals that can be extracted from TMR patients, potentially allowing them to more easily control multi-functional prosthetic limbs. I recommend accepting this paper with minor revisions, mainly in expanding the explanation and justification of choices made during the analysis.

Reviewer #3 (Report for the authors (Required)):

This case series of three participants with an upper extremity amputation evaluated the effects of a surgically implanted bio-interface with a high density micro-electrode array implanted within a reinnervated muscle with the ultimate goal of understanding motor unit behavior and controlling prosthetic limbs.

This work holds the potential to further develop the precision and usefulness of prosthetic limbs. Further, it advances our understanding of motor unit function which will be useful to understand muscle disease and injury and measure the effectiveness of therapeutic interventions.

Suggestion to improve the paper:
Discuss micro-electrode array sampling of motor units and the potential consequences of not recording all motor units in a muscle. What might be lost by not sampling all motor units? Might this impact the precision of prosthesis control?

Christopher D. Ingersoll, PhD
University of North Carolina - Chapel Hill

Version 1:

Decision Letter:

Dear Professor Farina,

Thank you for your revised manuscript, "Separation of Neural Drives to Muscle from Transferred Polyfunctional Nerves using Implanted Micro-electrode Arrays", which has been seen by the original reviewers. Despite our efforts, we didn't manage to get a response by Reviewer #3, but you can find find at the end of this message the reports of the other two experts, and you will see that the reviewers acknowledge the improvements to the work and only Reviewer #1 raises a few additional technical criticisms that we hope you will be able to address.

As before, when you are ready to resubmit your manuscript, please upload the revised files, a point-by-point rebuttal to the comments from all reviewers, the reporting summary, and a cover letter that

explains the main improvements included in the revision and responds to any points highlighted in this decision.

As a reminder, please follow the following recommendations:

*Please submit your revised manuscript in a word doc or LaTeX format.

* Clearly highlight any amendments to the text and figures to help the reviewers and editors find and understand the changes (yet keep in mind that excessive marking can hinder readability).

* If you and your co-authors disagree with a criticism, provide the arguments to the reviewer (optionally, indicate the relevant points in the cover letter).

* If a criticism or suggestion is not addressed, please indicate so in the rebuttal to the reviewer comments and explain the reason(s).

* Consider including responses to any criticisms raised by more than one reviewer at the beginning of the rebuttal, in a section addressed to all reviewers.

* The rebuttal should include the reviewer comments in point-by-point format (please note that we provide all reviewers will the reports as they appear at the end of this message).

* Provide the rebuttal to the reviewer comments and the cover letter as separate files.

We expect that you will be able to resubmit the manuscript within 12 weeks of receiving this message. If this is the case, you will be protected against potential scooping. Otherwise, we will be happy to consider a revised manuscript as long as the significance of the work is not compromised by work published elsewhere or accepted for publication at *Nature Biomedical Engineering*.

We look forward to receive a further revised version of the work. Please do not hesitate to contact me should you have any questions.

Best wishes,

Valeria

__
Dr Valeria Caprettini
Senior Editor, *Nature Biomedical Engineering*

____
Reviewer #1 (Report for the authors (Required)):

The authors have done a good job of addressing my concerns. I have only a few minor comments remaining.

1: “Moreover, the information content provided by implanted systems is much greater…
Therefore, in our view, there is no need to demonstrate... we have now explained in the Discussion the reasons for the information provided and for the lack of comparisons with other methods (lines 515-523). “
The comparison is not quite as simple as the authors suggest, and in any case, there is a cost benefit trade-off when one considers implementing implanted electrodes as opposed to surface electrodes (increased technical complexity, high spatial sampling vs. broader coverage, differences in surgery, and in the longevity of the implants, etc.). I think a paragraph addressing these topics would be warranted.

2: “On the other hand, we agree that we did not discuss these results sufficiently in the Discussion and we have therefore now added considerations on the physiological values in the revised Discussion (lines 548-557).”
How many EMG recordings were the 111 units decomposed from?
Was the feedback or task complexity different for TMR3 and 4? If not, these seem unlikely explanations.

5: “Low cosine similarity implies high orthogonality and therefore high separability of each time-varying activation. Average values of cosine similarity across tasks of each reinnervated muscle were 0.05 ± 0.03, 0.06 ± 0.02, 0.08 ± 0.08 and 0.12 ± 0.07, respectively.”
Of course, random noise is orthogonal and would also have low cosine similarity.
It would be good to show high similarity for a corresponding within-task measure.

“Finally, note that if the model of projection of synaptic input is the one we have postulated, the neural drives are mixed in linear instantaneous mixtures in the ensemble of spike trains. In these conditions, NNMF would identify the exact mixing matrix. “

I agree the NNMF is capable of finding this mixing matrix. The correspondence to tasks, however, is not a given. You have nicely demonstrated that this correspondence also occurs. My question is simply whether it might be further improved by a supervise method. I am not suggesting you implement something, but you might speculate on this point in the discussion.

8: “With this term we do not refer to the spatial organization of the muscle into distinct volumes/compartments. This has been clarified in the revised manuscript (Lines 565-572).”
I do not understand how the term “functional compartmentalization” is useful. Your description is of motor units that are distributed randomly throughout the muscle. Your analytical approach has the means to classify them functionally, but why refer to them as “compartmentalized”? It only seems to add confusion.

9: “In the sentence pointed out by the reviewer we meant that the presence of shared motor units and of covariation between motor unit activations is an indication that the motor unit space can be explained by a lower-dimensional manifold without losing significant information.”
My concern was a simpler (and minor) one. If you are referring (as I assume) to a manifold that you compute from available data (as opposed to a hypothetical construct), it MUST be of equal or lower dimension than the number of recorded units, which determines the embedding space of the manifold. Your sentence implies that this is not a given (“the presence of shared motor units between tasks indicated…“

Reviewer #2 (Report for the authors (Required)):

In their revised manuscript, the authors addressed reviewer comments by justifying thresholds and assumptions made during their original analysis. Primarily, the choice of a 70% presence threshold for active motor units across tasks prior to dimensionality reduction was shown to be valid by repeating the analysis with all motor units included and finding the the latent space to be of the same dimension. This addressed my concern about 'biasing' the latent space towards readily distinguishable signals. The authors also included a concrete quantification of the separation of tasks in the latent space via the cosine similarity measure. These results support the claims made about identifying distinct tasks with the lower dimensional embedding.

The authors have addressed my comments; I have no remaining questions.

Version 2:

Decision Letter:

Dear Prof Farina,

Thank you again for submitting to *Nature Biomedical Engineering* your revised manuscript "Separation of Neural Drives to Muscle from Transferred Polyfunctional Nerves using Implanted Micro-electrode Arrays". The manuscript has been seen by Reviewer #1, whose reports you will find at the end of this message. You will see that the reviewers appreciate the work, and that they raise a few additional clarifications that we hope you will be able to address.

As before, when you are ready to resubmit your manuscript, please upload the revised files, a point-by-point rebuttal to the comments from all reviewers, and a cover letter that explains the main improvements included in the revision and responds to any points highlighted in this decision.

As a reminder, please follow the following recommendations:

* Please submit your revised manuscript in a word doc or LaTeX format.

* Clearly highlight any amendments to the text and figures to help the reviewers and editors find and understand the changes (yet keep in mind that excessive marking can hinder readability).

* If you and your co-authors disagree with a criticism, provide the arguments to the reviewer (optionally, indicate the relevant points in the cover letter).

* If a criticism or suggestion is not addressed, please indicate so in the rebuttal to the reviewer comments and explain the reason(s).

* Consider including responses to any criticisms raised by more than one reviewer at the beginning of the rebuttal, in a section addressed to all reviewers.

* The rebuttal should include the reviewer comments in point-by-point format (please note that we provide all reviewers will

the reports as they appear at the end of this message).

* Provide the rebuttal to the reviewer comments and the cover letter as separate files.

We expect that you will be able to resubmit the manuscript within 6 weeks of receiving message. If this is the case, you will be protected against potential scooping. Otherwise, we will be happy to consider the next version of the manuscript as long as the advance of the work is not compromised by work published elsewhere or accepted for publication at *Nature Biomedical Engineering*.

We look forward to receive a further revised version of the work. Please do not hesitate to contact me should you have any questions.

Best wishes,

Valeria

__
Dr Valeria Caprettini
Senior Editor, *Nature Biomedical Engineering*

____
Reviewer #1 (Report for the authors (Required)):

The authors have responded well to all of my concerns, but slight clarification (and typo correction) might be useful related to point 3. The intent of my comment was to suggest computing a ceiling for the comparison that the authors present. However, I realize now that really was not appropriate in this context. The authors' response is fine. However, in the course of convincing myself of this, I made the following observations.

"We then quantified the degree of separability between each pair of time-varying activations in H for each reinnervated muscle using the cosine similarity measure, explained in Section 4.9. There is no mention of cosine similarity in 4.9, which instead, uses a coefficient of determination between the shapes of two MUAPs determined by spike-triggered averaging, together with a different measure of the spatial overlap of MUAPs on separate contacts. The reference to 4.9 should be corrected.

Cosine similarity is referred to in passing in 4.11, in the context of the application of NNMF. I found this section a bit difficult to wade through, partially because it switches between reference to latent variables, signals, and factors, all of which I believe are meant to be synonymous. This section ends with the comment, "The cosine similarity between pairs of latent factors is used to quantify the similarity between latent factors… Consine (sic) similarity is computed as the pairwise dot product, divided by the norm of each." I am mildly curious why cosine similarity was a more appropriate metric than correlation or coefficient of determination. If there is an interesting reason, you might give it together with the definition. Also, on line 1159, I noticed this typo: "Each raw of Hi is a latent variable…"

Version 3:

Decision Letter:

Dear Prof Farina,

Thank you for your revised manuscript, "Separation of Neural Drives to Muscle from Transferred Polyfunctional Nerves using Implanted Micro-electrode Arrays". Having consulted with Reviewers #1, I am pleased to write that we shall be happy to publish the manuscript in *Nature Biomedical Engineering*.

We will be performing detailed checks on your manuscript, and in due course will send you a checklist detailing our editorial and formatting requirements. You will need to follow these instructions before you upload the final manuscript files.

Please do not hesitate to contact me if you have any questions.

Best wishes,

Valeria

—
Dr Valeria Caprettini
Senior Editor, *Nature Biomedical Engineering*

____
Reviewer #1 (Report for the authors (Required)):

I hadn't actually expected to re-review this submission. It's in good shape.

Version 4:

Decision Letter:

Dear Prof Farina,

I am happy to inform you that your manuscript, "Implanted micro-electrode arrays in reinnervated muscles allow separation of neural drives from transferred polyfunctional nerves", has now been accepted for publication in *Nature Biomedical Engineering*.

Over the next few weeks, the figures will be checked for production quality, the text edited to ensure that it conforms to house style, and the manuscript typeset.

Our Articles are published about 40 days after the acceptance date (we recommend that you inform your institutional press office of this timeframe), and you will be notified of the actual publication date a few days in advance. Articles can be published any working day of the week, and are pushed live shortly after 10 am London time.

Publishing agreement. You will be asked to digitally sign a publishing agreement (grant of rights). After the signed publishing agreement has been received, the proofs of the article will be sent to you for review. If you have any queries during the production process, or you cannot meet the requested deadline for returning the proofs, please contact rjsproduction@springernature.com.

Nature Biomedical Engineering is a Transformative Journal. Authors may publish their research with us through the traditional subscription access route, or make their paper immediately open access through payment of an article-processing charge. More information about publication options is available.

You may need to take specific actions to comply with funder and institutional open-access mandates. If the work described in the accepted manuscript is supported by a funder that requires immediate open access (as outlined, for example, by Plan S) and your manuscript was originally submitted on or after January 1st 2021, then you should select the gold OA route. Authors selecting subscription publication will need to accept our standard licensing terms (including our self-archiving policies), and these will supersede any other terms that the author or any third party may assert apply to any version of the manuscript.

Acceptance of your manuscript is conditional on agreement, by all authors, with both our media embargo and confidentiality and pre-publicity policies. In particular, you may arrange your own publicity of the Article (for instance, through your institutional press office), as long as you ensure that journalists strictly adhere to the media embargo.

To assist you in disseminating the work, as soon as the Article is published you will be able to take advantage of the Springer Nature SharedIt initiative to generate a unique shareable link to the Article that will allow anyone (with or without a subscription) to read it. Recipients of the link who are subscribers will also be able to download and print the PDF.

Thank you for having submitted this work to *Nature Biomedical Engineering*.

Best wishes,

Valeria

—

Dr Valeria Caprettini
Senior Editor, *Nature Biomedical Engineering*

Open Access This Peer Review File is licensed under a Creative Commons Attribution 4.0 International License, which permits use, sharing, adaptation, distribution and reproduction in any medium or format, as long as you give appropriate credit to the original author(s) and the source, provide a link to the Creative Commons license, and indicate if changes were made.
In cases where reviewers are anonymous, credit should be given to 'Anonymous Referee' and the source.
The images or other third party material in this Peer Review File are included in the article's Creative Commons license, unless indicated otherwise in a credit line to the material. If material is not included in the article's Creative Commons license and your intended use is not permitted by statutory regulation or exceeds the permitted use, you will need to obtain permission directly from the copyright holder.
To view a copy of this license, visit https://creativecommons.org/licenses/by/4.0/

"Separation of Neural Drives to Muscle from Transferred Polyfunctional Nerves using Implanted Micro-electrode Arrays" (nBME-24-3438)

We thank the reviewers for the insightful comments and their time.
The original manuscript was modified in view of the reviewers' feedback.
Below 1) we summarize the main changes to each section of the manuscript; and 2) we provide detailed answers to the reviewers' comments.

Attached docs
Revised manuscript: "nBME-24-3438_revised.pdf"
Diff file, where changes in the manuscript are highlighted in colors: "nBME-24-3438_diff.pdf" file
Reporting summary: "nBME-24-3438_reporting_summary.pdf"
Cover letter: "nBME-24-3438_cover_letter.pdf"

1) SUMMARY OF REVISIONS

- We have clarified fundamental differences between the current work and Farina et al. 2017 and, as a consequence, we have explained why we did not include comparisons to traditional methods to extract motor intent information from TMR muscles.
- We have clarified the core new concept of synergistic behavior between motor neurons receiving common input.
- We have clarified our first hypothesis "the recorded neural activity of a reinnervated muscle could be modelled by a set of latent factors, with the number of factors being equal to or smaller than the number of measured tasks."
- As the reviewers stated, the results of the NNMF analysis indeed needed better quantification. We have now used the cosine similarity measure to quantify the "separability" between tasks for each reinnervated muscle. This provides a previously missing quantitative analysis.
- We have clarified the term "functional compartimentalization", referring to the organization of motor units into clusters/groups based on the common input they receive.
- We have demonstrated (and further discussed) that our motor unit inclusion criteria did not skew the results towards already separated signals. We have indeed repeated the NNMF analysis to estimate the neural manifold for TMR1 when including all motor units decomposed for each recorded tasks (i.e., without applying the exclusion criteria).
- We have explained why participants performed slightly different tasks. This was based on the participants' specific reinnervation profile, and ability to sustain or not long muscle contractions.
- We have discussed micro-electrode array sampling of motor units, the potential consequences of not recording all motor units in a muscle and the potential impact on precision of prosthesis control

2) ANSWER TO REVIEWERS

When addressing a reviewer's comment, we indicate in our answer the line numbers where corresponding changes were implemented in "nBME-24-3438_diff.pdf".

2.1) TO REVIEWR #1

1. **I started reading this paper, expecting a relatively straightforward analytical approach to maximize the information available following TMR. While this would've been similar to an earlier paper from the senior author (which they cited) the comparison to the results of those surface recordings could've been useful. However, except for the passing reference and citation, there was no such comparison made, nor any direct comparison to the information available from TMR patients by more traditional methods. The paper also contains analyses presumably meant to address basic science questions, some of them hypothesis driven, and others purely descriptive. Unfortunately, I found it rather difficult to take away any high-level conclusions from either theme. Better focus on a few central points could make this study more accessible and compelling.**

   In Farina et al. 2017, the concept of human-machine interface based on decoding the neural activity of reinnervated motor units, was first introduced. The focus of the work was then on mapping the motor unit activity, decomposed from surface EMG signals recorded for different tasks of the missing limb, into control signals using pattern recognition and musculoskeletal modeling. In that work, a 64-channel surface EMG grid was placed on each reinnervation site of participants. For example, in patient T1 of that previous study, data were recorded from 6 reinnervation sites to achieve an 11-gestures classification.

   Our present work differs substantially from that earlier study, and thus, was not intended for a direct comparison. One of the key differences is the use of acutely implanted microelectrode arrays, with a translational view for chronic implants. Compared to surface arrays, implanted ones would provide a better solution for patients, since these would eliminate issues of electrode stability over time due to electrode displacement and sweat, among other factors. Moreover, the information content provided by implanted systems is much greater than that of surface EMG, as demonstrated by both previous analyses as well as evident from the current study. This is because in surface EMG, the layers of biological tissue between the sensors and source of EMG signals act as a low-pass filter, significantly diminishing the bandwidth of the recorded signals. Previous analyses have shown, for example, a substantially greater number of identified motor units for a similar number of channels when decomposing surface and intramuscular EMG signals (Grison et al., IEEE TNSRE 2025). In our present study, we used only one implant (not six grids in six different reinnervation points, as in Farina et al., 2017) to decode a number of tasks similar to that in Farina et al., 2017. Therefore, in our view, there is no need to demonstrate that intramuscular micro-electrode arrays provide recordings with greater and more reliable information than surface EMG grids. This is very well established.

   Having said that, more importantly, in our study we aim to separate individual neural drives for tasks of the missing limb from a single micro-electrode array. The concept is that of decoding multiple functions from a single-muscle reinnervation with a polyfunctional nerve. This is achieved by recording the neural activity at high resolution using intramuscular electrodes, and by analyzing the common input to motor units active for different tasks. For this purpose, we observed the behavior of individual motor units, computed their short-term synchronization, and proposed a conceptual model of common input to reinnervated muscles which also explains why we can achieve such separation of neural drives. We finally directly proved that we can indeed identify the neural drives by factorization of motor unit spike trains (we do not map the neural activity from multiple reinnervation sites to motor classes as in Farina et al., 2017). In summary, we leverage the properties of hyper-reinnervation from a single transferred

nerve to extract multiple neural drives from a single reinnervation site and give proof of concept of a novel bio-interface that potential provides multiple control signals.

To reply to the comment “nor any direct comparison to the information available from TMR patients by more traditional methods**”**, as said above, we did not include a direct comparison to traditional methods for decoding motor intention from TMR muscles. Specifically, comparison with motor unit features extracted by surface EMG decomposition, as in Farina et al., 2017, would just be a comparison of decomposition accuracy of signals recorded by intramuscular microelectrode arrays and surface grids, but it is already well-known that intramuscular decomposition yield a greater number of motor units with greater accuracy with respect to surface decomposition (see, for example, Grison et al., IEEE TNSRE 2025, now also cited in the present manuscript). Comparison with global EMG classification (one of the traditional methods) is also not appropriate in our opinion since we do not provide classification results using the implanted systems. Rather, we provide a characterization of the motor unit behaviour, an analysis of their common input, and a factorization of their smoothed discharges (which confirms our findings regarding the common input to reinnervated motor units). This information cannot be obtained from classic EMG classification, and therefore a comparison would not be possible.

Because the points highlighted above are indeed important, we have now explained in the Discussion the reasons for the information provided and for the lack of comparisons with other methods (*lines 515-523*).

2. **Section 2.2.2 Describes the normality, mean firing rate, and CoV of the MU recordings, comparing the latter two measures across implants and to normally innervated muscle. The authors found no real patterns and did not return to these results in the discussion. It is not clear to me what they add.**
   Analysis of motor unit properties in reinnervated muscles was carried out to assess whether these were in the physiological range even after reinnervation before conducting further analysis. There is currently no other work providing a detailed physiological analysis of reinnervated motor units and we believe it is useful for the scientific community, especially in view of using motor unit activity to directly control prostheses. If the reviewer thinks the section is too extensive, we propose to reduce it to a couple of paragraphs and add a more detailed analysis in the supplementary material. On the other hand, we agree that we did not discuss these results sufficiently in the Discussion and we have therefore now added considerations on the physiological values in the revised Discussion (*lines 548-557*).

3. **Much is made of the presence of task-specific and task-independent motor units, the logic for which I find a bit difficult to follow, given that this distinction is entirely dependent on the choice of tasks. Motor units were also categorized with respect to strength of pairwise cross correlation, a measure of common input. When they compared the two measures, the authors concluded that, “common synaptic input appeared to be distributed across both task specific and shared motor units recruited for each task. This suggests that synergistic behavior between motor units is preserved even after reinnervation.” It is hard for me to understand how the authors draw this final conclusion, unless they are simply arguing that some common drive remains after**

reinnervation, not that it is unchanged.

We agree with the reviewer's comment, and we have rephrased the sentence in the revised manuscript. We did not mean to claim that the same synergistic behaviour as in physiologically innervated muscles is preserved.

Hug et al. 2023 recently proposed that common input is not limited to motor neurons belonging to the same pool (i.e., innervating the same muscle), nor necessarily to two or more entire pools of motor neurons. Common input can be projected to subgroups of motor neurons across multiple pools (i.e., to multiple fractions of motor neuron pools) with no direct association to the innervated muscles. This organization of common input is referred to as a synergistic structure since the multiple groups of neurons receiving common input act in combination to express motor behaviour. The fact that we detected correlated activity among motor units active for a task and that some of these motor units were active also during other tasks (shared units), implies that multiple groups of motor neurons receiving common input determine multiple tasks. This is the concept of synergistic activation of groups of neurons with weights that depend on the task. This is an important consideration since, on the one hand, it confirms a control strategy similar to that observed in physiologically-reinnervated muscles, and, on the other hand, the current experimental setup provides further evidence for the motor neuron synergistic theory stated by Hug et al., 2023. Indeed, a core concept of that theory is that the common input is not projected to pools of neurons (i.e., it is not directed to all motor units innervating a muscle) but to groups of neurons that might innervate a fraction of a muscle or fractions of multiple muscles. Thus, the CNS is not controlling muscles (muscles are irrelevant in this view). In our experimental conditions, we are in a situation where muscle fibres of a single muscle are reinnervated by motor neurons (previously innervating different muscles of the upper-limb) and these motor neurons receive multiple common inputs that functionally organise them into compartments within a single muscle. The CNS therefore controls the motor neurons without specific adaptations to the fact that, after TMR, they project on the same muscle vs a projection to multiple muscles in physiological conditions. Importantly, the structure of common inputs (number and projections to motor neurons) can be fully identified from the decoded spike trains of the motor units by factorization analysis, as we have done in this study. Since the above concepts are very important and should be clarified better to the reader, we have now expanded this part of the text and provided the above explanations (*lines 427-430*). We hope this is clearer.

4. **"We tested the hypothesis that the recorded neural activity of a reinnervated muscle could be modelled by a set of latent factors, with the number of factors being equal to or smaller than the number of measured tasks."**

   This is not a hypothesis, just standard dimensionality reduction.

   The statement that the number of factors is equal or smaller than the number of motor units may appear as obvious since we applied a dimensionality reduction method to the motor unit space. However, here we hypothesized something different, i.e. that the number of factors extracted from the motor unit space (and that reconstructs the motor unit space with high accuracy) is equal or smaller than the number of tasks. This is a less obvious hypothesis since the number of motor units may be much greater than the number of tasks. The dimensionality of the motor unit space is determined by the total number of motor units active (and decomposed) across the different tasks of the missing limb. A motor unit may be recruited across different tasks. For example, for TMR1, the motor unit space dimension is 31. From the

common input analysis, we know that motor units active for a task receive a portion of common input and independent inputs. Common inputs to multiple motor units cause common low frequency oscillation in their neural activity. In practice this means that there is redundancy in the motor unit space – the variability in the motor unit space can be explained by a lower dimensional manifold (neural manifold) embedded in the motor unit space. However, we do not know a priori if the dimensionality of the neural manifold corresponds to the number of tasks. The dimensionality could be larger than the number of tasks if motor units received multiple inputs, or the participant was executing multiple tasks simultaneously, for example. The dimensionality would be lower than the number of tasks if motor units recruited for a task received the same input as motor units recorded for another task (shared motor units). We therefore think this is an important, and testable hypothesis. We aimed to estimate the number of neural drives (common inputs) given to the motor units recruited across all the tasks, and we assessed if such dimensionality was equal or smaller than the number of recorded tasks. We did this by estimating the optimal dimensionality of the neural manifold embedded in the motor unit space (NNMF was repeated multiple times with different number of latent factors/imposed dimensionality of the neural manifold) and checking whether this matched the number of recorded tasks. This has been clarified in the manuscript (*lines 441-448*).

5. **"We then evaluated the hypothesis that each dimension of the lower-dimensional latent space (i.e., neural manifold) captured key patterns of neural activity that corresponded to a recorded movement of the phantom limb."**
This is a testable hypothesis, and the result shown in Fig 6 is particularly impressive with respect to the separation between tasks. It is more or less, what I expected the paper to be about. However, this result is never quantified, and appears to be from a single nerve of a single participant. How does the information provided from this type of intramuscular recording compare to what can be obtained with high density surface electrodes? Would it be worth the added complexity? Also, unless I am missing something, NNMF, which is unsupervised (unlike dPCA), would not necessarily be expected to find a projection that separates tasks like this. This might be worth some comment in the discussion, particularly given the suggestion about the use of these signals for control. Might some further de-mixing step be necessary in some cases?

Regarding the comparison to surface EMG, please see our answer at point 1).

We agree these results of the NNMF analysis needed better quantification. In the results (and methodology) we have now added a paragraph where we use the cosine similarity measure to quantify the "separability" between tasks for each reinnervated muscle (*lines 495-509*). The cosine similarity is computed for each pair of time-varying activations in H (rows of H), see figure below. Low cosine similarity implies high orthogonality and therefore high separability of each time-varying activation. Average values of cosine similarity across tasks of each reinnervated muscle were 0.05 ± 0.03, 0.06 ± 0.02, 0.08 ± 0.08 and 0.12 ± 0.07, respectively. All tasks of TMR1,2 exhibited high separability. In fact, all tasks had at least one task-specific motor unit (independent). The higher standard deviation for TMR3 and TMR4 indicates that some tasks could not be separated optimally. As discussed in the manuscript, Pinky Flexion and Ulnar deviation in TMR3, and Hand Open in TMR4 had no independent motor units. This corresponded to a higher cosine similarity between H4 and H5 in TMR3, H2 and H3,

H3 and H6 in TMR4. We have now provided this numerical characterization for all cases in the revised

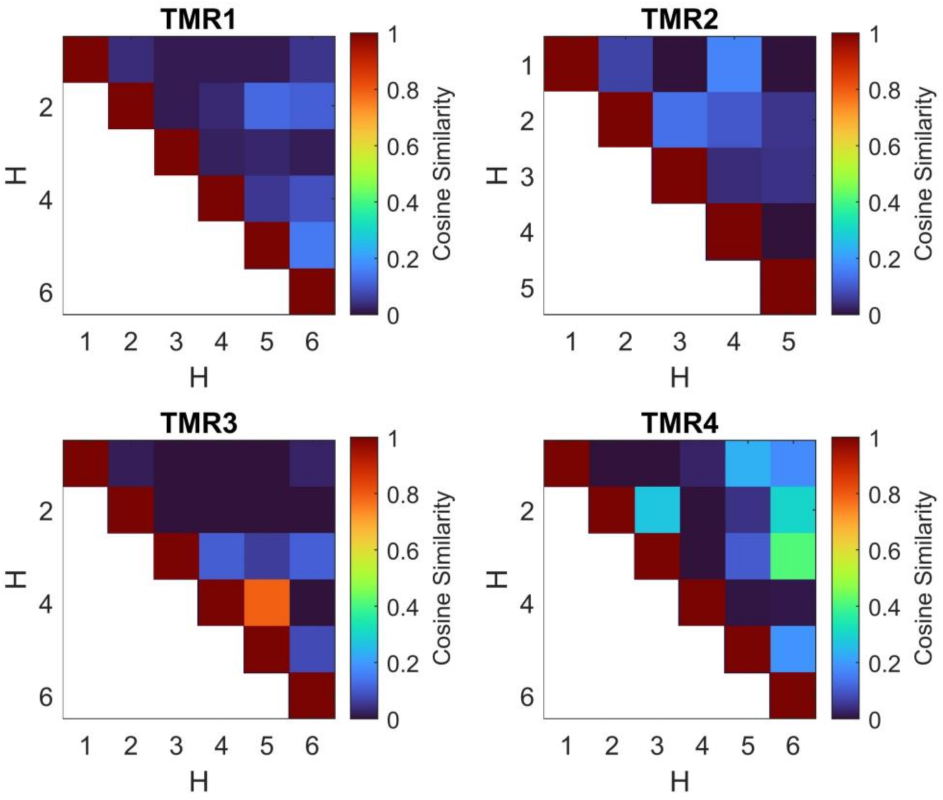

manuscript.

In response to “appears to be from a single nerve of a single participant”: we report the results from all reinnervated muscles, we do not consider a single nerve/single reinnervated muscle only. While the results from all reinnervated muscles are discussed in the text (*Lines 474-509*), due to the limited space, the corresponding plots for the motor unit and latent spaces of TMR2,3,4, as in Fig6 for TMR 1, are included as Extended Data Fig 7,8,9. Now, these results are also fully quantified, as discussed above.

Finally, note that if the model of projection of synaptic input is the one we have postulated, the neural drives are mixed in linear instantaneous mixtures in the ensemble of spike trains. In these conditions, NNMF would identify the exact mixing matrix.

6. “**All tasks in TMR1 and TMR2 had at least one task-specific motor unit, suggesting that the dimensionality of the neural manifold should correspond to the number of tasks.**”
   I do not follow this logic, particularly if this is meant to describe a general concept, as opposed to a comment about this particular experimental outcome.
   We slightly amended the cited sentence into “All tasks in TMR1 and TMR2 presented at least one task-specific motor unit, suggesting that the dimensionality of the neural manifold could not be lower than the number of tasks.” (*Lines 474-476*). This is because each task was characterised by at least the activity of one unit, different from others. We have demonstrated that we could obtain a control signal for each task that had at least an independent motor unit. All tasks in TMR1 and TMR2 had at least an independent motor unit, meaning that dimensionality of the neural manifold would be not smaller than

the number of recorded tasks.

7. “**Overall, a total of 19 out of the 23 recorded tasks in four reinnervated muscles could be decoded from the proposed biointerface.”**
I do not understand this statement. At the very least, it must refer to classification as opposed to decoding. However, these 23 tasks were distributed across multiple nerves from multiple participants. The best this could represent is separate classifications among much smaller numbers of tasks.
We agree with this comment and have modified the sentence accordingly to avoid giving the impression we recorded and separated 23 tasks from a single reinnervated muscle. We have modified the revised manuscript accordingly:
“In summary, a distinct control command could be extracted for each recorded task of reinnervated muscles 1 and 2. Distinct control commands were identified for 4 out of 6 tasks in reinnervated muscle 3 and 5 out of 6 tasks in reinnervated muscle 4.” (*Lines 493-497*)
8. **“We thoroughly analyzed the functional roles of motor units consistently recorded across multiple task repetitions and observed distinct clustering based on their functional contributions…These findings demonstrate that reinnervation via polyvalent nerves leads to a functional compartmentalization within the muscle.”**
This seems to refer to spatial (“compartmentalization within the muscle”), as opposed to functional clustering, although I don’t think anything like that was demonstrated. Did I miss it? If not, please clarify this statement.
We have seen that motor units are heterogeneously distributed across different channels of the micro-electrode array (meaning that muscle fibers belonging to different motor units are intermingled). As shown in Fig.2, motor units may have different morphology – they may span few (Fig.4) or multiple channels (Fig.2). This means that motor units recruited for a task are not necessarily spatially compartmentalized (i.e., they can be distributed in a large muscle portion and not only in a small muscle portion, which would be a spatial compartment). On the other hand, we have demonstrated that there are groups/clusters of motor units recruited for a particular task of missing limb, meaning that motor units recruited for the same task received common input. These clusters/groups of motor units are defined purely based on a task/functional role. We refer to this “clustering” as “functional compartmentalization”. With this term we do not refer to the spatial organization of the muscle into distinct volumes/compartments. This has been clarified in the revised manuscript (*Lines 565-572*).
9. “**The covariation between motor units recruited for a task, and the presence of shared motor units between tasks indicated that the underlying neural activity was confined to a neural manifold of dimension lower than the number of identified motor units.”**
Does this refer to a manifold you can compute from available data, or to a hypothetical underlying manifold? The former must be the case. The latter, clearly not, but the data can’t really address that point. Can this statement be clarified?
We are referring to a neural manifold embedded in the space defined by the number of motor units recruited across the tasks of a reinnervated muscles (e.g., Fig 6-a is the motor unit space for TMR1; Fig.6-d is the neural manifold (the latent space embedded in the motor unit space) obtained using NNMF). By simply observing the neural activity of motor units in Fig.6-a, we see there are common

oscillations between neural signals. This is because motor units may receive common inputs, as discussed in Section 2.4.1, which cause instances of covariation between the neural activity of motor units. As a result, due to such covariation, the space defined by all recruited motor units (Fig 6-a) is redundant; we can estimate a lower-dimensional space (neural manifold) embedded in the space of the motor units (Fig 6-d).

In the sentence pointed out by the reviewer we meant that the presence of shared motor units and of covariation between motor unit activations is an indication that the motor unit space can be explained by a lower-dimensional manifold without losing significant information.

10. **"Targeted Muscle Reinnervation (TMR) involves the transfer of polyfascicular nerves, which previously innervated muscles of the missing limb, to the motor nerves of surgically denervated target muscles [3].**"
    At least, as originally described in the cited publication, the anastomosis was to denervated muscle, not to a nerve.
    This has been fixed in the revised manuscript (*Line 83*)
11. **"…as well as real-time visual feedback of the reinnervated muscle EMG activity (as recorded by the micro-electrode array),"**
    Some indication here, of how that was done. 1D display of a single channel?
    A description has been added at *lines 191-193* of the revised manuscript. Given a task and we selected two channels of the micro-electrode array that resulted in the maximum EMG amplitude when the participant was performing the task. We obtained a bipolar signal from the two monopolar signals recorded by the two selected channels and used it as visual feedback.
12. **"TMR nerve transfer surgery: a polyfascicular nerve that previously innervated multiple upper-limb muscles (dotted purple line) was transferred into a spare target muscle (continuous light purple line)." The reference to purple lines appears to be a mistake.**
    This has been fixed in the revised manuscript (*Line 1321*)
13. **"In an exemplary task repetition,"**
    I suspect you mean "exemplar" here and elsewhere, unless you are intentionally showing examples that are not representative of typical behavior.
    This has been fixed in the revised manuscript (*Line 1363*)
14. **"…participant P3 isometrically contracts muscle TMR4"**
    Why would this be considered an isometric contraction?
    We agree that this is incorrect and have removed the term in the revised manuscript
15. **"…with a pulse-to-noise ratio >30 dB [24]."**
    Define this metric here.
    In the revised manuscript we have added a descriptive definition of the metric ("a signal-to-interference metric introduced to quantify how accurately a motor unit signal can be distinguished given the signal background noise" at *line 837-839*), the mathematical formulation and its derivation can be found in Holobar et al., 2014.
16. **"We further investigated the distribution of ISIs by computing the histogram of the inter-spike intervals with 1-ms bins (Fig.2-f): ISIs had either skewed or multimodal distributions. How does this and analysis differ from that of the previous paragraph? Why both?"**
    This was to provide examples of such non-normal distributions of ISIs by referring to figure 2-f. In the

revised manuscript we have removed this sentence and referenced Figure 2-f. We could remove panel f from Figure 2 and only retain the description in section 2.2.2, if the reviewer suggests so.

17. **“…median discharge rate (MFR). Change either the name or the abbreviation, so that they match”**
    In the revised manuscript, “Mean discharge rate” instances were changed to “mean firing rate”
18. **“Despite large variability, the MFR were within the physiological range reported for voluntary contractions of physiologically innervated muscles”**
    Why is this summary here, rather than in the previous paragraph where MFR was discussed?
    The variability in discharge rate is quantified using the coefficient of variation of interspike intervals, introduced and computed in the paragraph above the sentence highlighted by the reviewer. We believe the sentence is in the correct position, but “Despite large variability” has been rephrased to clarify that we refer to the coefficient of variation.
19. **“The mean and standard deviation values of MFR and CoV per each task of the reinnervated muscles are reported in Tab.1 of the Supplementary Material.”**
    Same question here. This paragraph is no longer about these values.
    In the revised manuscript, we removed the sentence at *lines 300-302* and added the following after discussing both MFR and CoV at *lines 289-291*: “The average task-specific values and the average values of MFR and CoV per muscle (across the different tasks) are reported in Tab.1 of the Supplementary Material.”
20. **“…common input was 34.00°æ10.09%, 25.76°æ18.80%, 18.77°æ15.35%, and 31.04°æ9.07%. I would suggest that you drop one or two significant figures when reporting numbers for which the standard deviation is nearly the same magnitude of the mean.”**
    Fixed in the revised manuscript. We dropped one significant figure.
21. **“The observed motor unit behavior enabled us to propose a conceptual model illustrating how the central nervous system encodes movements post-reinnervation (Fig.6-c). This is a reference to 6-e, not 6-c“**
    Fixed in the revised manuscript.

2.2) TO REVIEWR #2

1. **How was the decision to exclude motor units not present in at least 70% of the tasks was reached and how that threshold was chosen**
   In amputees we do not have a ground truth telling us whether they are performing a particular task. We can, however, rely on consistency of motor units' recruitment across repetitions. For this reason, we asked participants to repeat the same task a few times. 70% was an arbitrary value, chosen to retain a reasonable number of repetitions for different participants. The first participant could sustain longer contractions, so we defined the protocol to include 4 reps of 4 (ramp up) + 10 (constant) + 4 (ramp down) seconds. The other participants had more difficulties in maintaining the contractions, so we decided to reduce the contraction time and increase the number of repetitions to ensure more frequent breaks. We used a threshold of 70%, which led to retaining motor units that were consistently recruited in 3 out of 4 reps (0.7*4 reps = 2.8 -> 3 reps) for P1 and 4 out of 6 reps (0.7*6=4.2 > 4 reps) for P2 and

P3. Having said that, because the choice of 70% threshold may appear arbitrary, we have also repeated the analyses including all motor units (see next reply).

2. **Given that the paper is an analysis of the separability of motor unit activation according to movement, could the removal of some fraction of the signals not potentially bias the results towards already-separate signals?**
   Spurious motor units, firing only a few times such as those removed according to the 70% reps consistency threshold, would have not changed the results since they contribute to little variability in the data. Motor units shared across tasks and consistently active across repetitions (which we retain) are those affecting the signal separability. To support our claim, we have now repeated the NNMF analysis to estimate the neural manifold for TMR1. In this case the motor unit space consisting of ALL the motor units recruited during the recorded tasks, even those active for < 70% of the repetitions of tasks, remained of the same dimension as when excluding the motor units active for less than 70% of the trial.

   In the figure below we report the smoothed motor unit spike trains constituting the motor unit space. When comparing this “complete” motor unit space (A) to the one reported in Fig.6-a (B) it can be noticed that Pinky Flexion and Abduction have motor units active in less than 70% of repetitions that we initially discarded in (B) from the analysis. In (A), all motor units are tracked to ensure that motor units shared across tasks are identified. We estimate the optimal dimensionality of the neural manifold embedded in the motor unit space (A) using NNMF. (A) and (B) are both explained by a neural manifold of dimension 6 (see figure below). Thus, removing spurious motor units did not impact the neural manifold analysis and the signal separability. In response to point 5) of Reviewer 1 we have included the use of the cosine similarity measure to quantify the “separability” between tasks of each reinnervated muscle. The cosine similarity is computed for each pair of time-varying activations in H (rows of H). Low cosine similarity implies high orthogonality and therefore high separability of each time-varying activation (in H). Average value of cosine similarity across tasks of TMR1 in the two cases were not significantly different. This additional analysis has been included in the revised manuscript.

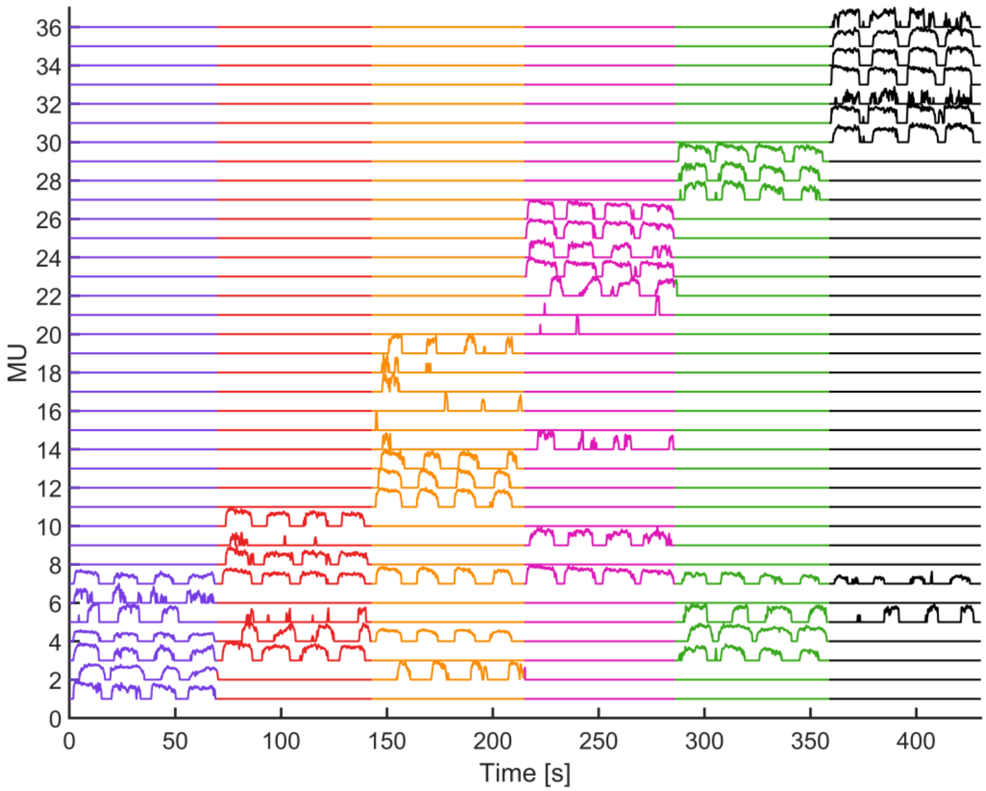

MU
Time [s]
0
2
4
6
8
10
12
14
16
18
20
22
24
26
28
30
32
34
36
0
50
100
150
200
250
300
350
400

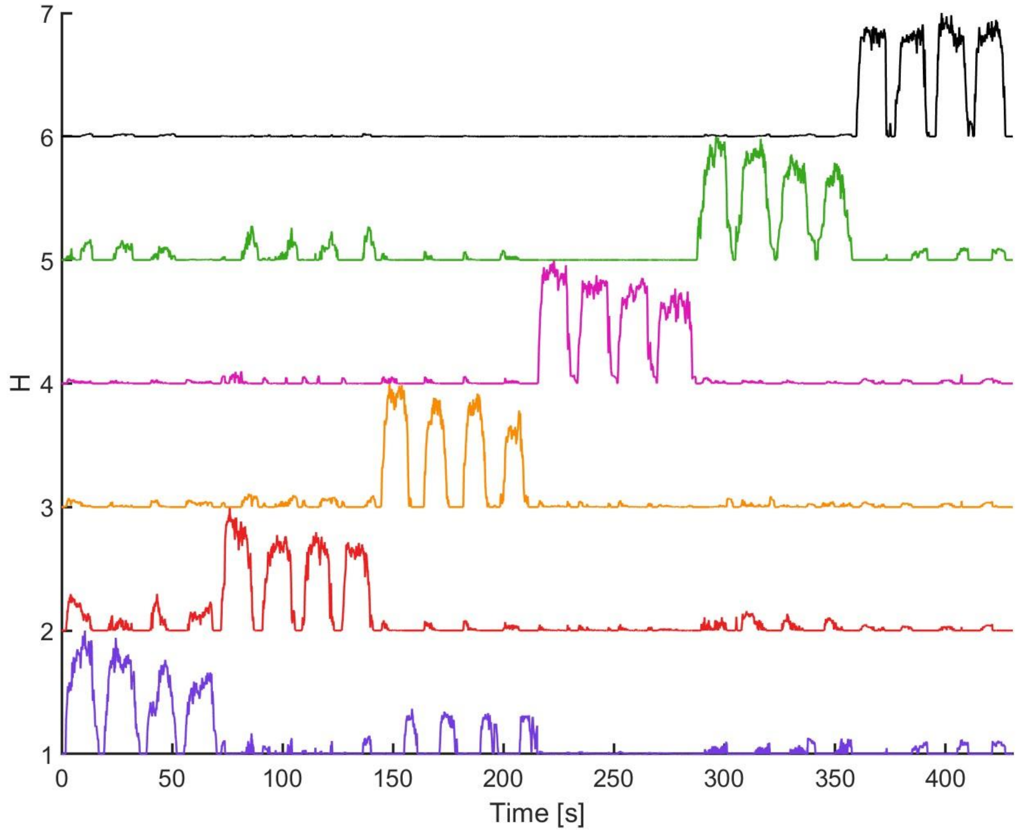

Due to the lack of space, we have not included these two figures in the manuscript. We have justified the choice of the threshold value (70%) used in the exclusion criteria by describing the analysis detailed above in the text (*Lines 830-836*).

3. **It would be helpful to know how many total motor units were identified before trimming to the used 111; this would indicate how consistent the overall activation patterns were**
   In total, across the 4 reinnervated muscles and all the recorded tasks, 21 MUs were removed. In TMR1, considering all tasks, 9 units were removed (1.5 ±1.9 on average per task). In TMR2, 12 units were removed (2.4 ± 1.5 on average per task). No units were removed in TMR 3. In TMR4, only one motor unit was removed (0.2±0.4 on average per task). This information has been included in the manuscript (*Lines 237-239*)
   Below, an example for TMR1 Pinky Flexion. A total of 12 motor units were decomposed across the task repetitions. A total of 4 motor units (MU number 7,8,10,11) were then discarded according to our criteria.

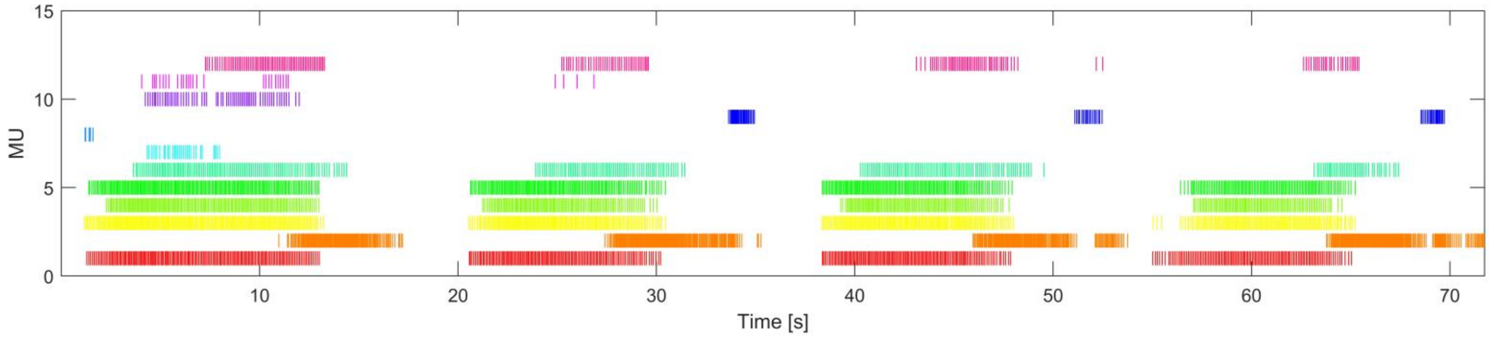

4. **Further, performing dimensionality reduction with NNMF should not have required the exclusion of those signals. As they were inconsistent, NNMF could have found a latent space of the same dimension using the (b) criteria of the paper (the coefficient of determination method) without a meaningful increase in reconstruction error. Demonstrating that the dimension of the latent space remained unchanged or increased only slightly with all motor units would be strong support justifying their exclusion.**
In the previous answer, we have demonstrated that the dimension of the latent space remained unchanged in TMR1.

5. **Why were the ramping protocols varied between each participant? The most MU were found in TMR1, with P1 having the longest isometric hold. Could this have influenced the results?**
The experimental protocol was 'tailored' to participants depending on their specific reinnervation profile, and capabilities. Tasks were defined based on reinnervation profile, and the participant's ability to perceive the missing limb performing a particular task (hence increasing the likelihood of the participant actually performing the task). Depending on individual condition, we defined the length of the contraction and the number of repetitions. Participant 1 could easily sustain 18-sec long contractions, whereas this was not feasible for the other two participants. We therefore decided to have shorter contractions, to allow more frequent breaks, and we increased the number of repetitions. We have clarified this in the Methodology section (*Lines 744-745*).

The reason why we observed more motor units in TMR1 (P1) may be due to a range of reasons, and it is difficult to tell. Considering the participants' details in Table 1, it could be that the level of training and use of prosthetics could be key factors. P1 uses his prosthesis for 15 hours a day, every single day of the week. In contrast, P2 and P3 said they wear their prosthesis on average twice a week.

6. **TMR1/2 and TMR3/4 present different results in a few places (section 2.2.2 and the size of the latent spaces). The discussion section doesn't address that these groupings occur in patients with different amputation levels, even with the same reinnervated target muscle (for TMR1/TMR3). Are there other mechanisms that could've impacted motor unit recruitment – like the presence of proprioceptive or sensory afferents from the residual upper arm in P1?**
In our study, due to the limited size of the population sample (4 reinnervated muscles from 3 participants), it is not possible to systematically and rigorously evaluate the effect of participant's characteristics (such as level of amputation) on the observed results. In our preliminary analysis (not

included in the manuscript, as explained above), we have not observed any significant difference due to the different level of amputation or the type of transferred nerve.

In P1 we did not record from any residual muscle in the upper limb, but from the pectoralis minor, which is reinnervated by the ulnar nerve. We can neither quantify the impact of an impaired sensory system on our results (effect on motor unit recruitment), nor know if such impairment was similar across participants.
We believe that two main factors may have had an impact on our results: (i) the participant's ability to perform a task, which may be correlated to frequency of prosthesis usage during everyday life; (ii) the inability to record the neural information for a task due to micro-electrode array placement and pick-up. Considering the first point (i), it can be observed that P1, who uses its prostheses every day for about 15 hours, had the most motor units decoded and we could extract a control signal for each task. P2 and P3 use the prosthesis only a couple of days a week, which may have had an impact on their ability to imagine and execute some tasks. This may have been a reason why we could not find any independent motor unit for Ulnar Deviation in TMR3, for example. One other reason may be (ii) that no motor units recruited for that task were in the pick-up area of the sensor. To summarize, it becomes difficult to clearly identify the factor that could have affected the variability in our results.

7. **Section 4.4 describes the signal quality assessment and exclusion criteria – how many signals were used from each electrode array?**
   We report the average root mean square of the baseline noise across all channels for each micro-electrode array. All channels recorded high quality signals, and we did not have to discard any channel.
8. **The text or figure captions refer to dotted lines or shaded areas in some of the figures that aren't present – please check for consistency between the final figures and text (purple lines in figure 1, shaded blue in figure 2, blue color in figure 5)**
   This has been fixed in the revised manuscript.
9. **A few sentences are hard to understand, especially around line 169 and line 1111. It appears that some phrases may have been combined, and transition phrases were dropped**
   While we are unsure if the reviewer is referring to a specific sentence, below we have rephrased the paragraphs at lines 169 and 1111 of the original manuscript. Corresponding changes were made in the revised manuscript

   <u>Rephrased paragraph at line 169 of original manuscript:</u>
   First, the nerve is divided into fascicles without prior knowledge of the functional role of each. This may result in fascicles, and therefore RPNI units, that do not provide a usable control signal. Second, detecting EMG signals from each RPNI unit remains unfeasible outside a laboratory setting. Third, the number of fascicles - and thus the potential number of independent signals — is limited by the need to attach each fascicle to a muscle graft. Finally, these fascicles are sutured to a small segment of muscle not respecting the neuromuscular entry zone and thus run the risk of not making any functional connections. In such cases, RPNI units are lost while the surgery results in permanent damage of the nerve (*Lines 123-134*).

   <u>Rephrased paragraph at Line 1111 of original manuscript:</u>

d'Avella et al. [48] and Clark et al. [38] selected the minimum number of factors beyond which an additional factor increased the variance accounted by the latent components less than 5%. The variance accounted for was computed as (1 − SSE)/
SST, where SSE (sum of squared errors) was the unexplained variance and SST (total sum of squares) was the total variance. Authors of [49] identified the optimal number of factors by computing the mean square error (MSE) curve obtained from linearly fitting the R2-curve with an increasing number of factors. The optimal number of latent factors corresponds to the maximum number
of factors for which the MSE remains lower than 10^-5. A threshold value 5 × 10−4 was set in [50]. In our study, NNMF is applied for values of I ranging from 1 to 15. The coefficient of determination R2 is computed as in Muceli et al. [37]. The MSE values
are reported for completeness. The number of latent factors is determined using the R2-curve as in d'Avella et al. [48] and Clark et al. [38] *(Lines 949-965).*

2.3) TO REVIEWR #3

Discuss micro-electrode array sampling of motor units and the potential consequences of not recording all motor units in a muscle.

1. **What might be lost by not sampling all motor units?**
2. **Might this impact the precision of prosthesis control?**

Increasing the density and number of channels of the micro-electrode array, as well as the array size, would increase the number of motor units that are decomposed and consequently the accuracy of the motor pool representation (Caillet, Arnault H., et al, 2023). However, even when increasing the number of channels, it may not be possible to sample the activity of all motor units innervating a muscle since motor units are distributed in a large area and some may have only few fibers.
In our study, the inability to sample all motor units means losing the neural information regarding some tasks of the missing limb. For example, we could not extract an independent signal for ulnar deviation in reinnervated muscle 3. Only one motor unit, shared across other task of the same reinnervated muscle, could be accurately decomposed. This may be a case where either the neural activity for ulnar deviation was not in the pick-up range of the sensor, or the participant was not able to execute the task.

We have demonstrated that a single independent motor unit is sufficient to disentangle the control signal for a task. However, a limited sample of the neural activity for a particular task will limit the precision in estimating the neural drive for that task.
In practice, this may impact the precision of prosthesis control in terms of the participant's ability to finely control a particular task/movement. Precision in prosthesis control is affected by several factors (socket, mechanics of prosthesis, design of control strategy, etc) that are not only limited to the human-machine interface. Precision of neural drive reconstruction may not necessarily imply precision of control. For this reason, we aim to address these important research questions in our future work.

We have added a paragraph about these important points (*Lines 586-590*).

"Separation of Neural Drives to Muscle from Transferred Polyfunctional Nerves using Implanted Micro-Electrode Arrays" (NBME-24-3438A)

We thank all the reviewers for the insightful comments and their time.
The original manuscript was modified in view of Reviewer#1's feedback.
Below, 1) we summarize the main changes to the manuscript; and 2) we provide detailed answers to Reviewer#1's comments.

Attached docs

Revised manuscript: "nBME-24-3438A_revised.pdf"
Diff file, where changes in the manuscript are highlighted in colors: "nBME-24-3438A_diff.pdf" file
Reporting summary: "nBME-24-3438A_reporting_summary.pdf"
Cover letter: "nBME-24-3438A_cover_letter.pdf"

1) SUMMARY OF REVISIONS

- We extended the Discussion to address the differences between surface and intramuscular EMG in more detail.
- We have clarified that TMR3 and TMR4 are from the same participant 3, as opposed to TMR1 and TMR2 from participant 1 and 2. Thus, we explained the reason for discrepancies in the number of decomposed motor units and their firing behavior across the reinnervated muscles of different participants.
- We explained the methodology to minimize the presence of errors and random noise in decomposed spike trains
- We included a small paragraph explaining how the manifold estimation may be improved by extending the non-negative-matrix factorization cost function with the regularization term of a supervised method, and why this may be relevant in our future developments.
- We removed the terms "functional compartmentalization"
- We clarified that, in our study, explaining the motor unit space with a manifold of dimension lower (not equal) than the motor unit space dimension is not a given, but a testable hypothesis.

2) ANSWER TO REVIEWER#1

When addressing a reviewer's comment, we indicate in our answer the line numbers where corresponding changes were implemented in "nBME-24-3438A_diff.pdf".

1. **"Moreover, the information content provided by implanted systems is much greater... Therefore, in our view, there is no need to demonstrate... we have now explained in the Discussion the reasons for the information provided and for the lack of comparisons with other methods (lines 515-523). "**
   The comparison is not quite as simple as the authors suggest, and in any case, there is a cost benefit trade-off when one considers implementing implanted electrodes as opposed to surface electrodes (increased technical complexity, high spatial sampling vs. broader

coverage, differences in surgery, and in the longevity of the implants, etc.). I think a paragraph addressing these topics would be warranted.

We agree that a better explanation of this important issue is needed and, accordingly, we have added three paragraphs in the revised manuscript (Lines 729-741,866-868, 871-874).

"With respect to invasive solutions, surface high-density arrays allow broader muscle coverage non-invasively, with simpler deployment and higher patient acceptance, but at the cost of reduced spatial selectivity, lower bandwidth due to tissue filtering, and increased variability over time due to replacement of the electrodes at each use or changes in skin conditions. Moreover, while surface arrays can support motor unit decomposition, their stability and the number of reliably trackable units remain inferior to intramuscular recordings (Grison et al., 2025). Intramuscular recordings indeed provide higher resolution, signal fidelity, and access to deep muscle fibers, enabling more accurate decoding of motor intent (Salminger et al., 2019). High-density multi-channel intramuscular micro-electrode arrays, as those used in our present work (Muceli et al., 2018), enabled the recording of multi-source observation (intramuscular recording at each channel) for direct motor unit decomposition (Muceli et al., 2018; Grison et al., 2025)."

"However, as opposed to surface electrodes, invasive systems introduce technical complexity, especially for a possible chronic implant application, and potential risks associated with longevity of implants (e.g., encapsulation, migration, or device failure)."

And "Future developments aiming to minimise the invasiveness of implanted systems while maximising coverage and longevity could mitigate technical complexity, simplify the deployment and increase patient acceptance."

2. **"On the other hand, we agree that we did not discuss these results sufficiently in the Discussion and we have therefore now added considerations on the physiological values in the revised Discussion (lines 548-557)."**
   How many EMG recordings were the 111 units decomposed from?
   Was the feedback or task complexity different for TMR3 and 4? If not, these seem unlikely explanations.

The number of EMG recordings (tasks and repetitions per reinnervated muscle of participants) is detailed in Section 4.1 and summarised in Table 1. In total, we recorded 23 tasks, 6 in TMR1, 5 in TMR2, 6 in TMR3 and 6 in TMR4. In Figure 3, we show the number of motor units (out of the total 111) recruited for each task, for each reinnervated muscle.
The visual feedback was obtained from intramuscular recordings in the same manner for each reinnervated muscle (Line 970). The overall task complexity surely varied across participants, due to individual characteristics (amputation, nerve transfer matrix for TMR, prosthesis usage, ...). TMR1 and TMR2 are reinnervated muscles from participants 1 and 2, respectively. TMR 3 and TMR 4 are from the same participant 3 (TMR3 Pectoralis minor reinnervated by Ulnar nerve; TMR4 Latissimus dorsi reinnervated by Radial nerve). Therefore, across participants and reinnervated muscles, there

may be differences in the complexity of generation of the neural drive for each task, which depends on the state of the muscle and reinnervation and is affected by the participant's ability to execute the task. Moreover, while the micro-electrode arrays were inserted with the same methodology (insertion area is identified as the most myoelectrically active part of the muscle observed through clinical examination), some motor units recruited for a task may not be in the pickup volume of the electrodes. These two factors impact the number of recruited motor units that can be decomposed per task. The firing behaviour of available motor units is affected by the participant's ability to perform the task and maintain a stable contraction (solely guided by visual feedback on the myoelectric activity of the reinnervated muscle).
We have added these considerations in the revised manuscript (Lines 764-771).

3. **"Low cosine similarity implies high orthogonality and therefore high separability of each time-varying activation. Average values of cosine similarity across tasks of each reinnervated muscle were 0.05 ± 0.03, 0.06 ± 0.02, 0.08 ± 0.08 and 0.12 ± 0.07, respectively."**
   Of course, random noise is orthogonal and would also have low cosine similarity.
   It would be good to show high similarity for a corresponding within-task measure.
   The reviewer raises a good point. Random noises would also be orthogonal, and thus a similarity measure based on orthogonality would not necessarily imply high quality, meaningful separability of the time-varying activations. In general, noise may reduce the cosine similarity index. However, in our case, the activations were obtained from factorization of motor unit activities, which are high-quality estimates obtained with the following steps: 1) we have inspected and reported the quality of recorded signals (average root mean square of the baseline noise, when the muscle is at rest) showing high quality low noise recordings (Lines 285-286) and absence of noisy channels, which guarantees an accurate automatic decomposition; 2) we have manually inspected each output motor unit spike train with the spike-sorting interactive software EMGLAB to ensure the correctness of decomposition (identify wrongly assigned or missing spikes) and ensure noise was not identify as a separate latent component (i.e., motor unit spike train); 3) we inspected the motor unit action potential MUAP to ensure they had physiological characteristics (amplitude, morphology of action potential), and the average discharge properties (median firing rate, coefficient of variation) to ensure values were within the expected physiological ranges; 4) the accuracy of motor units spike trains decoding was evaluated using with a Pulse-to-Noise-Ratio (mean of detected spiking activity divided by the mean baseline of estimated source in dB). Motor unit spike trains with PNR lower than 30 dB (Holobar et al., 2014) were removed ("Screening of motor units" in Methodology section). Overall, these results ensure high quality of spike trains that were used within the manifold analysis. In this condition, we exclude a major effect of noise on the extracted activations.

   The manifold analysis using NNMF is done per reinnervated muscle, considering the motor unit space defined by all motor units decomposed for the recorded tasks. We used the cosine similarity to evaluate the orthogonality between the latent components of the obtained neural manifold, as a proxy of quality of signal separability. This similarity measure is therefore between tasks, not within

tasks, as we do not obtain multiple latent factors for a single task (dimensionality analysis). If the reviewer means that noise may be affecting the latent components equally, and suggests a within-task analysis to assess whether the contribution to high orthogonality is given by neural drive to motor units and not by random noise, we would like to point out the following:
In Figure 6-c, we show the non-negative matrix of normalized contributions of motor units (of all tasks) to latent components. Considering, for example, the latent component 1, which in this case refers to Wrist Flexion, it can be observed how motor units 1 and 2, with a weight above 0.6, mainly contribute to the latent signal, while the remaining motor units either do not contribute to the latent component or only weakly. Among the "highly contributing" motor units, the independent ones are those contributing towards a low similarity score.

4. **"Finally, note that if the model of projection of synaptic input is the one we have postulated, the neural drives are mixed in linear instantaneous mixtures in the ensemble of spike trains. In these conditions, NNMF would identify the exact mixing matrix. "**
I agree the NNMF is capable of finding this mixing matrix. The correspondence to tasks, however, is not a given. You have nicely demonstrated that this correspondence also occurs. My question is simply whether it might be further improved by a supervise method. I am not suggesting you implement something, but you might speculate on this point in the discussion.

In the current work, we demonstrated that NNMF was capable of unmixing the neural signals into latent components that corresponded to the recorded tasks in an unsupervised manner. Using an unsupervised method was needed since we did not use the number of tasks as a priori information, but we tested if the dimensionality of the estimated manifold was equal to the number of recorded tasks.

However, NNMF is an unsupervised method, it does not use any labelling of training data, and it solely relies (in its simpler version) on minimizing the reconstruction error to solve a non-convex optimization problem. Thus, less dominant patterns in the activity of motor unit spike trains may not be captured because less relevant towards high reconstruction accuracy. However, these patterns may be relevant in view of maximising the number of signals associated with tasks that can be extracted from muscles reinnervated by a polyvalent nerve. Moreover, NNMF is prone to different inconsistency and redundancy errors that may nevertheless have a low impact on the reconstruction accuracy. The suitability of the cost function used for NNMF (does accurate data approximation achieved by NNMF translate into an accurate signal separation?), and the incorporation of constraints to the optimization problem, may be further explored in future work. One approach might be the use of a semi-/supervised method, where a priori labelled motor units (with respect to a task) are used in the optimization problem. The NNMF cost function could be extended with a regularization term that incorporates the cost function of supervised methods. This may ensure further consistency and robustness of the NNMF output.

As the reviewer suggests, we have added a small paragraph on this in the revised manuscript (Lines 811-814).

5. **“With this term we do not refer to the spatial organization of the muscle into distinct volumes/compartments. This has been clarified in the revised manuscript (Lines 565-572).” I do not understand how the term “functional compartmentalization” is useful. Your description is of motor units that are distributed randomly throughout the muscle. Your analytical approach has the means to classify them functionally, but why refer to them as “compartmentalized”? It only seems to add confusion.**

   To avoid unnecessary confusion based on this terminology, we have decided to remove the term “functional compartmentalization” in the revised manuscript (Lines 788-793), as the reviewer suggested.

6. **“In the sentence pointed out by the reviewer we meant that the presence of shared motor units and of covariation between motor unit activations is an indication that the motor unit space can be explained by a lower-dimensional manifold without losing significant information.”**
   My concern was a simpler (and minor) one. If you are referring (as I assume) to a manifold that you compute from available data (as opposed to a hypothetical construct), it MUST be of equal or lower dimension than the number of recorded units, which determines the embedding space of the manifold. Your sentence implies that this is not a given (“the presence of shared motor units between tasks indicated…“

   We agree with the reviewer. Of course, a manifold would necessarily have an equal or smaller dimensionality than the original data’s one. However, in our sentence, revised in the previous revision we meant only that “lower” dimensionality is not a given, but a testable hypothesis: “… presence of shared motor units between tasks indicated that the underlying neural activity was confined to a neural manifold of dimension lower than the number of identified motor units.” The manifold would necessarily be of dimension smaller or equal, but not necessarily of dimension smaller.

"Separation of Neural Drives to Muscle from Transferred Polyfunctional Nerves using Implanted Micro-Electrode Arrays" (NBME-24-3438B)

We thank Reviewer#1 for their time and thorough comments that helped us to further improve the manuscript. The original manuscript was modified in view of Reviewer#1's feedback.
Below, 1) we summarize the main changes to the manuscript; and 2) we provide detailed answers to Reviewer#1's comments.

Attached docs

Revised manuscript: "nBME-24-3438B_revised.pdf"
Diff file, where changes in the manuscript are highlighted in colors: "nBME-24-3438B_diff.pdf" file
Reporting summary: "nBME-24-3438B_reporting_summary.pdf"
Cover letter: "nBME-24-3438B_cover_letter.pdf"

1) SUMMARY OF REVISIONS

Two typos and the wrong section reference (identified by the reviewer) have been corrected. We spotted some other typos and fixed them. Section 4.11 has been revised to ensure consistency in terminology when referring to the latent variables of neural manifolds. We clarified why we chose to use cosine similarity instead of other correlation measures.

2) ANSWER TO REVIEWER#1

When addressing a reviewer's comment, we indicate in our answer the line numbers where corresponding changes were implemented in "nBME-24-3438B_diff.pdf".

1. **The authors have responded well to all of my concerns, but slight clarification (and typo correction) might be useful related to point 3. The intent of my comment was to suggest computing a ceiling for the comparison that the authors present. However, I realize now that really was not appropriate in this context. The authors' response is fine. However, in the course of convincing myself of this, I made the following observations.**
"We then quantified the degree of separability between each pair of time-varying activations in H for each reinnervated muscle using the cosine similarity measure, explained in Section 4.9. There is no mention of cosine similarity in 4.9, which instead, uses a coefficient of determination between the shapes of two MUAPs determined by spike-triggered averaging, together with a different measure of the spatial overlap of MUAPs on separate contacts. The reference to 4.9 should be corrected.

Thank you, the section we meant to reference was Section 4.11. This has now been corrected (Line 709).

2. **Cosine similarity is referred to in passing in 4.11, in the context of the application of NNMF. I found this section a bit difficult to wade through, partially because it switches between reference to latent variables, signals, and factors, all of which I believe are meant to be synonymous.**

We have revised Section 4.11 to ensure consistency. We have maintained the term latent "variables".

3. **This section ends with the comment, "The cosine similarity between pairs of latent factors is used to quantify the similarity between latent factors… Consine (sic) similarity is computed as the pairwise dot product, divided by the norm of each." I am mildly curious why cosine similarity was a more appropriate metric than correlation or coefficient of determination. If there is an interesting reason, you might give it together with the definition. Also, on line 1159, I noticed this typo: "Each raw of Hi is a latent variable…"**
   The correlation coefficient can be considered as the cosine similarity between centred variables/vectors. As a result, while the correlation coefficient is invariant to both shift and scaling of the variables, cosine similarity is invariant to scaling only. Because we wanted a metric that captured differences in the direction of the latent variable while disregarding differences in their magnitude, we chose the cosine similarity.
   We have reported this brief explanation in the revised manuscript (Line 1198).
   We have also corrected the two typos ("raw", "consine") for which we apologize.
